# Testing the Impact of Intensive, Longitudinal Sampling on Assessments of Statistical Power and Effect Size Within a Heterogeneous Human Population: Natural Experiment Using Change in Heart Rate on Weekends as a Surrogate Intervention

**DOI:** 10.2196/60284

**Published:** 2025-05-21

**Authors:** Severine Soltani, Varun K Viswanath, Patrick Kasl, Wendy Hartogensis, Stephan Dilchert, Frederick M Hecht, Ashley E Mason, Benjamin L Smarr

**Affiliations:** 1 Bioinformatics and Systems Biology Graduate Program University of California San Diego La Jolla, CA United States; 2 Shu Chien-Gene Lay Department of Bioengineering University of California San Diego La Jolla, CA United States; 3 Department of Electrical and Computer Engineering University of California San Diego La Jolla, CA United States; 4 Osher Center for Integrative Health University of California San Francisco San Francisco, CA United States; 5 Zicklin School of Business Baruch College New York, NY United States; 6 Halıcıoğlu Data Science Institute University of California San Diego La Jolla, CA United States

**Keywords:** physiological heterogeneity, physiological variability, sample size, wearable sensors, mHealth, biomedical science

## Abstract

**Background:**

The recent emergence of wearable devices has made feasible the passive gathering of intensive, longitudinal data from large groups of individuals. This form of data is effective at capturing physiological changes between participants (interindividual variability) and changes within participants over time (intraindividual variability). The emergence of longitudinal datasets provides an opportunity to quantify the contribution of such longitudinal data to the control of these sources of variability for applications such as responder analysis, where traditional, sparser sampling methods may hinder the categorization of individuals into these phenotypes.

**Objective:**

This study aimed to quantify the gains made in statistical power and effect size among statistical comparisons when controlling for interindividual variability and intraindividual variability compared with controlling for neither.

**Methods:**

Here, we test the gains in statistical power from controlling for interindividual and intraindividual variability of resting heart rate, collected in 2020 for over 40,000 individuals as part of the TemPredict study on COVID-19 detection. We compared heart rate on weekends with that on weekdays because weekends predictably change the behavior of most individuals, though not all, and in different ways. Weekends also repeat consistently, making their effects on heart rate feasible to assess with confidence over large populations. We therefore used weekends as a model system to test the impact of different statistical controls on detecting a recurring event with a clear ground truth. We randomly and iteratively sampled heart rate from weekday and weekend nights, controlling for interindividual variability, intraindividual variability, both, or neither.

**Results:**

Between-participant variability appeared to be a greater source of structured variability than within-participant fluctuations. Accounting for interindividual variability through within-individual sampling required 40× fewer pairs of samples to achieve statistical significance with 4× to 5× greater effect size at significance. Within-individual sampling revealed differential effects of weekends on heart rate, which were obscured by aggregated sampling methods.

**Conclusions:**

This work highlights the leverage provided by longitudinal, within-individual sampling to increase statistical power among populations with heterogeneous effects.

## Introduction

Statistical comparisons are vital to ascertaining meaningful differences between groups in the biomedical sciences. In a standard power analysis required by many funders, researchers may increase power by increasing the number of observations (n), which is typically achieved by increasing the number of participants [1,2]. Power analyses assume that observations are independent and identically distributed (IID). As a result, repeatedly measuring the same individual could lead to pseudoreplication and confound statistical analyses, as the longitude leads to both dependence of values on that individual, and dependence on the time of sampling. In biological systems, this IID assumption often does not hold. If there are unaccounted for, nonrandom sources of variance across observations, these sources may limit the increase in power achieved by larger sample sizes while obscuring small effects. Although variance is usually large in aggregated, population-level data, repeated sampling of data within individuals or within a specific time frame may reduce variance, improving statistical power [3-5].

Off-the-shelf wearable devices (eg, smartwatches and smart rings) can continuously measure physiological data such as heart rate (HR), a commonly integrated physiological measure, from the user [6]. As wearable devices have become more prevalent, so has the generation of longitudinal time series data of physiological measures captured by these devices. While some methods of data collection may yield IID data, individuals’ physiology and behavior heavily influence the distribution of measurements collected by wearable devices (eg, nonrandom lifestyle choices may cause one’s physiology on Saturday nights to look reliably quite different from Tuesday nights; [7-9]). As a result, time series data from wearable devices have proven useful for enabling in-depth monitoring of individuals’ physiology for applications such as early infection detection [10-13]. The newly available continuous, longitudinal, within-participant measurements for physiological samples afforded by wearable devices may prove useful for increasing confidence in measurements. Representative measurements are critical for applications such as latent class analysis, where individuals are grouped into distinct phenotypes based upon these values. Such data could be used to control for different sources of variance, thereby increasing statistical power and the detection of changes specific to subpopulations within clinical trials or research studies.

Variance in physiological or behavioral measurements may fall into 2 well-recognized categories: interindividual and intraindividual variability [14,15]. Interindividual variability refers to differences between individuals. For example, resting HR is known to have a wide interindividual variability, with the normal resting HR for an individual being anywhere from 50-90 beats per minute [16,17]. Intraindividual variability refers to changes within an individual’s physiology across time. An individual’s physiology can vary even on short timescales. For example, an individual’s HR will likely be higher when they are ill compared with when they are healthy, regardless of how their healthy baseline HR compares to anyone else’s; however, such intraindividual changes in HR have been found to be smaller contributors to overall variance than the changes across individuals [6,11-13,18]. Interindividual and intraindividual differences contribute structured changes (ie, the source of variance is nonrandom) in which the underlying distribution of these data may differ significantly between individuals and across time. Time series data from wearables allow for extensive repeated measurements of an individual’s baseline physiology to obtain a more precise estimate of what is normal for each individual under different circumstances. Such longitudinal sampling allows for an unprecedented level of statistical control for both interindividual and intraindividual physiological variability.

To investigate the contributions of these structured sources of variance in data, we analyzed a longitudinal dataset of HR data collected during sleep from the TemPredict study [19]. We use HR because it is a physiological measure known for its substantial inter- and intraindividual variability, making it an ideal candidate for investigating the contribution of different sources of variability in longitudinal data. By leveraging wearable devices, which offer continuous and precise measurements from single individuals over time, we can explore how variations across individuals and within individuals over time contribute to the overall physiological heterogeneity observed in the data.

In this study, we focus on understanding how controlling for both interindividual and intraindividual variability affects the statistical power in detecting study sample-level and individual-level physiological responses to weekends. We leverage weekends as a naturally occurring periodic event, which are expected to reliably change some individuals’ physiology due to lifestyle choices [7-9]. We specifically examine how these sources of variability influence the number of samples needed to reach statistical significance and the associated effect size at significance. By using the extensive and continuous data afforded by wearables, we investigate whether accounting for these different sources of variability allows us to better detect physiological responses to weekends, and how wearables enhance our ability to detect heterogeneity in individual-level physiological responses to weekends that might otherwise be masked with sparser traditional sampling methods.

## Methods

### Ethical Considerations

Mason et al [19] outlined initial participant recruitment in the University of California San Francisco institutional review board (#20-30408) approved this study on March 17, 2020; the United States Department of Defense Human Research Protections Office (#E01877.1A) also approved all study activities. All research was performed in accordance with relevant guidelines and regulations and the Declaration of Helsinki. All participants provided electronic, informed consent to participate in the study via a University of California San Francisco Qualtrics consent survey.

### Data and Device

The original cohort from [19] comprised 63,153 participants with available multimodal physiological data. For this particular study, we narrowed down the participant pool to 46,374 individuals with nightly physiological measurements acquired from a wearable device, the Oura Ring Gen2 (Oura Health Oy). The Oura Ring is a commercially available wearable device that may be worn on any finger continuously during daily activities in both wet and dry environments. The Oura Ring connects to the Oura App (available on the Google Play Store and Apple App Store) via Bluetooth. The Oura Ring calculates interbeat intervals using a 250 Hz photoplethysmogram signal during periods of sleep to compute HR. Previous work has externally validated the accuracy of HR measured by the Oura Ring [20]. Oura calculates HR measurements during sleep and yields an average HR during sleep (“sleep summary”). In addition to physiological data, participants self-reported their demographic information, including sex, age, race, geographical location, and educational level.

We used these sleep summary HR values spanning from January 6, 2020, to October 18, 2020, for a total of 322 nights (41 full weeks). During this timeframe, 24 participants had no HR data, reducing the number of participants to 46,350.

We excluded HR values of below 30 beats per minute or above 100 beats per minute as these values tend to be outside the bounds of normative HR [16,17]. This eliminated data from 3 individuals, reducing the number of participants to 46,349. We classified “weekend night” HR measurements as occurring on Friday or Saturday night and “weekday night” measurements as occurring on all other nights of the week. We ensured that all participants had at least 1 weekend night and 1 weekday night of data per each calendar week for which they have HR data. This reduced the number of participants to 46,217.

### Analysis Software

We used Python software ([21]; version 3.11.9; Python Software Foundation) for all analyses. This included the pycountry-convert package (version 0.7.2) for computing geographical cohort composition; the SciPy ([22]; version 1.13.1) and scikit-posthocs packages ([23]; 0.11.2) for conducting statistical analyses; and the cliffs-delta package (version 1.0.0) for calculating effect sizes.

### Interindividual and Intraindividual Variability in HR

To gauge the variation in resting HR between individuals compared to HR within individuals, we used a subset of HR data from the month of May, as it included the greatest number of individuals with complete data for the entire month. We found the SD of HR for each of the 16,010 individuals with complete data for May and plotted the distribution of SDs. To compare individual SDs to our sample aggregate from the month of May, we found the SD of HR across all the HR values from the 16,010 individuals for this month.

We gauged intraindividual variations in HR on the order of weeks to months by taking the median HR for each night across all individuals. Median computations ignored missing nightly values. We compared (1) weekly fluctuations in HR due to weekends and weekdays, and (2) seasonal fluctuations in HR by comparing HR data from January and February to March and April. We further split up individuals into quartiles based on the magnitude of the difference between their mean weekend and mean weekday night HR across all their available data to see how weekly HR fluctuations differ between individuals. We used the Mann-Whitney *U* test to compare weekly, seasonal, and quartile-dependent fluctuations in HR and reported Cliff *δ* [24] as a measure of effect size: we used Cliff *δ* here and in subsequent analyses to measure effect size between potentially non-Gaussian distributions. To better illustrate weekend-weekday HR differences, we use daily-level data using time-delay embeddings with a lag of 7 days from two individuals: (1) with greater HR on weekends, and (2) with greater HR on weekdays.

### Sampling Methods

#### Cohort‑Wide Methods

We initially created a dataset on which we could test the efficacy of different cohort-wide methods, that is, sampling methods that use HR data from across our entire dataset of 46,217 individuals. To create this dataset, we randomly sampled one weekend night and one weekday night HR value from a random calendar week for each individual. We did this once for all 46,217 individuals in the dataset, resulting in 46,217 pairs of weekend night and weekday night HR values. We created 1000 of these datasets for 1000 simulated “runs” of each below sampling methods.

#### Random Sampling

To simulate random, cross-sectional sampling (ie, not accounting for variability due to either individuals or time), we scrambled the original cohort-wide dataset so that it was no longer paired by individual or by week. We then iterated over each pair of HR samples, successively adding each pair to a Mann-Whitney *U* test; we used the Mann-Whitney *U* test due to the unpaired nature of the samples. We found the iteration at which *P*<.01. That iteration was equal to the number of pairs sampled at the point of statistical significance; we computed Cliff’s *δ* using sampled values up to and including that iteration. We include Multimedia Appendix 1 to help illustrate a few iterations of this and subsequent sampling methods.

#### Temporal Sampling

Temporal sampling controlled for variability due to time, thereby accounting for systematic fluctuations due to temporal factors such as seasonality. This sampling method kept HR values from the initial dataset paired by calendar week but scrambled the HR values across individuals to disrupt the individual-level pairings. Thus, this sampling method maintained only the paired-by-week nature of the original cohort-wide dataset. Due to the samples being temporally paired, we used a Wilcoxon Signed-Rank test rather than the Mann-Whitney *U* test to find the iteration at which *P*<.01. We computed Cliff *δ* at the point of significance.

#### Person-Matched Sampling

To account only for interindividual variability, we used person-matched sampling. We maintained the order of individuals as they were sampled in the original cohort-wide dataset, but instead of sampling a weekend-weekday HR pair from the same week, we randomly selected a weekend night HR sample from any point in time and a weekday night HR sample at any point in time. Due to the samples being paired by the individual, we used a Wilcoxon Signed-Rank test rather than the Mann-Whitney *U* test to find the iteration at which *P*<.01. We computed Cliff *δ* at the point of significance.

#### Temporal Person-Matched Sampling

This sampling method controlled for both interindividual and temporal variability across the study sample. Here, we used the original cohort-wide dataset without any modifications. Therefore, each weekend-weekday pair was from the same individual and the same calendar week. Thus, due to the samples being paired by both individual and time, we used a Wilcoxon Signed-Rank test rather than the Mann-Whitney *U* to find the iteration at which *P*<.01. We computed Cliff *δ* at the point of significance. Note that this sampling method still did not provide for repeated weeks within any individual. Each individual is still represented only by a single pair of weekend-weekday HR samples.

#### Within-Individual Methods

Here, we compared within-individual methods of sampling and the impact of controlling for temporality in different manners. In order to make the methods comparable to each other, we limited the number of weekend-weekday pairs per individual to however many valid weeks of HR data each individual has. Meaning, a week of data counted as “valid” if there was at least 1 weekend and weekday value from which to sample. Given that our data spans from January to October, each individual had anywhere from 1 to 41 weeks of data from which to sample for each sampling method.

#### Within-Individual Sampling

This method of sampling controlled for only interindividual variability by focusing on a single individual’s data. We separated the individual’s data into weekday and weekend nights. We iteratively randomly sampled weekend-weekday night HR pairs from these 2 pools, without replacement, until we reached the maximum number of valid weeks for each individual. At each iteration, we conducted a Wilcoxon Signed-Rank test and computed Cliff *δ*. Given that our sampling methods were probabilistic, a single run of sampling may not accurately represent an individual’s true weekend-weekday night HR difference; therefore, we conducted 100 runs per individual. Each run used the same procedure for finding points of significance and computing Cliff *δ*. We computed the median points of significance and Cliff *δ* over the 100 runs. For the median computations, we ignored runs that failed to reach significance. This was necessary, as statistical modes cannot be calculated on distributions that extend into infinity.

#### Within-Individual Temporal Sampling

This method of sampling controlled for intraindividual (ie, temporal) variability on the level of an individual. We selected a single individual and their data. At each iteration, we chose a random calendar week of their data and selected a random weekend-weekday night HR pair from that week until the set of valid weeks from which to sample was depleted. As in within-individual sampling, we used 100 runs of sampling for each individual. Likewise, we computed statistical significance using the Wilcoxon Signed-Rank test and Cliff *δ* over the 100 runs identically to the within-individual sampling method.

#### Within-Individual Sequential Sampling

Much like within-individual temporal sampling, this method controlled for both interindividual and intraindividual variability. However, we chose the weeks sequentially rather than randomly, beginning from the individual’s first calendar week of data. The rationale behind this method was that data that are temporally closer to each other are more alike than data separated by many weeks or months. This similarity may imply less variation in weekday night and weekend night HR distributions, leading to a smaller number of samples needed to reach significance. As in within-individual temporal sampling, we conducted 100 runs of sampling and used the Wilcoxon Signed-Rank test and Cliff *δ* over the 100 runs.

### Simulated Power Analysis

To contextualize the real-world benefit of within-individual sampling, we conducted a power analysis using simulated normal distributions. We varied effect size and statistical power to determine the number of samples required to detect a significant difference between 2 normal distributions. We used effect sizes ranging from 0.05 to 0.95 in increments of 0.05. We used statistical power thresholds ranging from 0.05 to 1.00 in increments of 0.05. We used these 2 known variables to calculate the number of pairs of samples required to detect a significant difference at a significance threshold *α*=.01 using both the Mann-Whitney *U* test and the Wilcoxon Signed-Rank test.

## Results

### Participant Characteristics

We outlined participant characteristics in Table 1. Our cohort consisted primarily of White individuals residing in either North America or Europe.

**Table 1:** Demographic characteristics of participants (N=46,217). Participants were able to self-identify as more than one race.

Demographic characteristic		Values
**Sex, n (%)**		
	Male	27,639 (59.8)
	Female	17,450 (37.8)
	Other	38 (<1)
	Undisclosed or unavailable	1090 (2.4)
**Age (years), n (%)**		
	18-19	97 (<1)
	20-29	4767 (10.6)
	30-39	11,825 (26.2)
	40-49	13,415 (29.7)
	50-59	9493 (21.0)
	60 years or older	5444 (12.1)
	Undisclosed or unavailable	1176 (2.6)
**Race, n (%)**		
	White	37,871 (83.9)
	Asian	2429 (5.4)
	Hispanic	2387 (5.3)
	South Asian	762 (1.7)
	Black	680 (1.5)
	Middle Eastern	558 (1.2)
	Native American	229 (<1)
	Native Hawaiian	147 (<1)
	Other	1847 (4.1)
	Undisclosed or unavailable	1090 (2.4)
**Geographical location, n (%)**		
	North America	28,969 (62.7)
	Europe	13,180 (28.5)
	Oceania	1395 (3.0)
	Asia	1177 (2.5)
	South America	189 (<1)
	Africa	106 (<1)
	Undisclosed or unavailable	1201 (2.6)
**Education, n (%)**		
	Less than a high school diploma	211 (<1)
	High school diploma or GED	1704 (3.7)
	Some college	4302 (9.3)
	Associate degree	1751 (3.8)
	Bachelor’s degree	16,110 (34.9)
	Master’s degree	13,506 (29.2)
	Professional degree	4143 (9.0)
	Doctorate	2594 (5.6)
	Undisclosed or unavailable	1896 (4.1)

### HR Shows Interindividual and Intraindividual Variability

**Figure 1:**
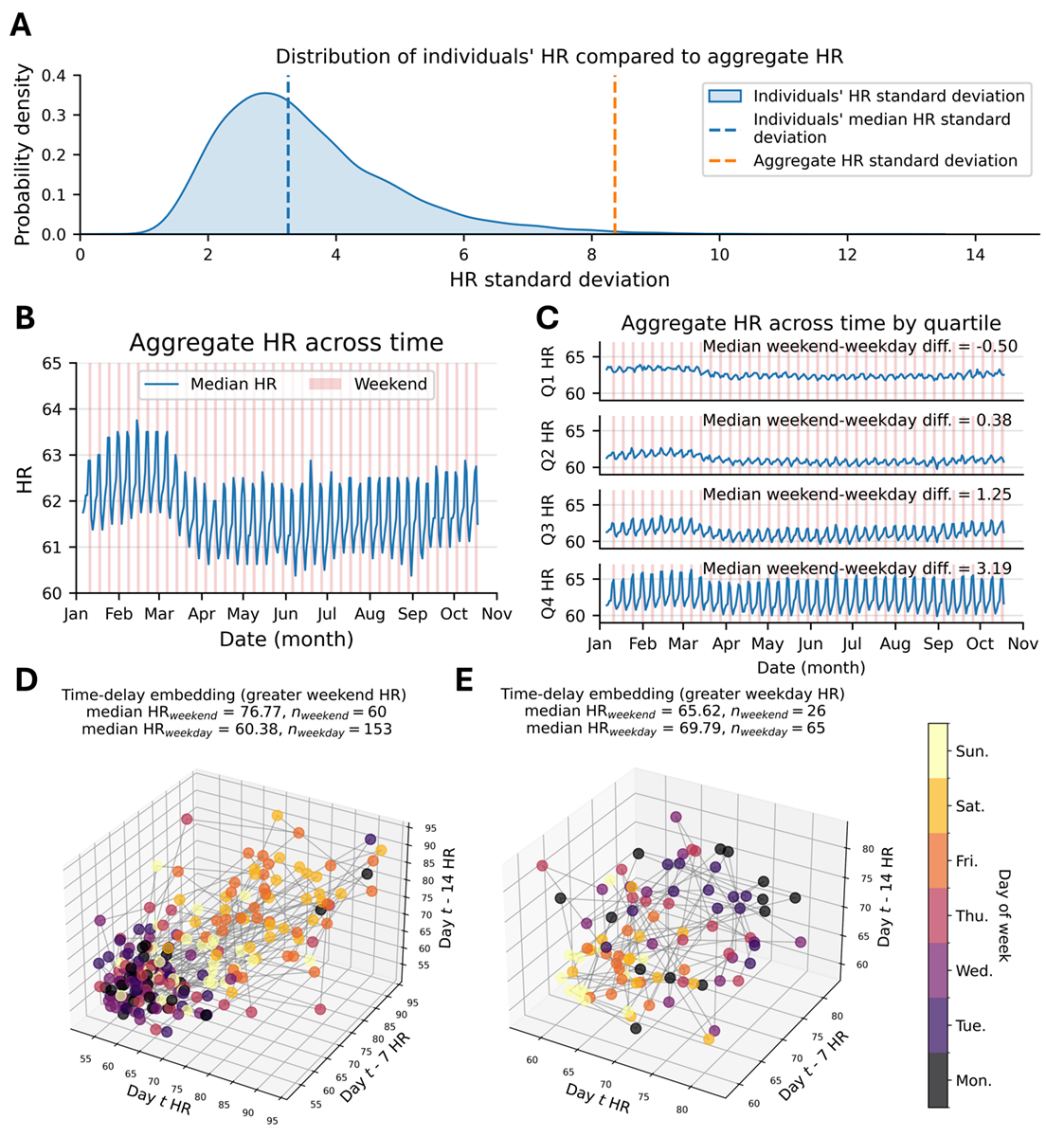
Variation in heart rate (HR) data across individuals, time, and conditions. (A) Comparison of individuals’ SD of HR compared with the aggregate SD of HR within the month of May. (B) Median daily HR across time (C) individuals from (B) were grouped into quartiles based on the difference in their mean weekend and mean weekday night HR. (D) Time-delay embedding of an individual with greater HR on weekends. (E) Time-delay embedding of an individual with greater HR on weekdays.

Individuals’ HR SD was substantially lower than that of the aggregate population in almost all cases (Figure 1A). We found that the aggregate population SD fell at approximately the 99th percentile of the distribution of individual-level SDs. We confirmed that median intraindividual variability was seasonal: The median HR from January to February was 62.375 (IQR 57.000-68.500) beats per minute (bpm), and HR from March to April was 61.625 (IQR 56.250-67.750) bpm (*P*<.001; *δ*=0.05; Figure 1B). We observed weekly-level intraindividual variability: We found that the median HR on weekend nights was 62.500 (IQR 57.000-68.750) bpm, which was significantly higher than the median value of 61.375 (IQR 56.000-67.375) bpm on weekdays (*P*<.001; *δ*=0.08; Figure 1B). We also confirmed the presence of significant heterogeneous population responses in weekend-weekday night HR differences: The median difference in HR on weekend nights compared with weekday nights across quartiles was 3.188 (IQR 2.438-4.625) bpm, 1.250 (IQR 1.000-1.562) bpm, 0.375 (IQR 0.250-0.625) bpm, –0.500 (IQR –1.000 to –0.188) bpm for quartiles 4, 3, 2, and 1, respectively (Figure 1C). The difference in weekend-weekday night HR varied significantly between quartiles (*P*<.001 for all comparisons between quartiles; Figure 1C). We illustrate time-delayed embeddings of a representative individual with greater weekend night HR (Figure 1D) and an individual with greater weekday night HR (Figure 1E).

### Pairs of Samples Required for Significance

#### Cohort‑Wide Methods

The median pairs of samples required to find a statistically significant difference between weekend night and weekday night HR at *P*<.01 was 428.05 (IQR 173.00-838.75; Figures 1A, 1C, and 1D). We also provide the mean (SD) for each sampling method at *P*<.01 in Multimedia Appendix 2, as well as the median (IQR) and mean (SD) for each sampling method at *P*<.05 in Multimedia Appendix 3. Temporal sampling similarly required 460.50 (IQR 186.75-839.00) pairs to reach significance for the same comparison (Figures 1A, 1C, and 1D). There was no significant difference between random and temporal sampling (*P*=.39). Person-matched sampling and temporal person-matched sampling required 3-4 times fewer pairs to reach significance at 133.00 (IQR 62.00-227.25) and 113.00 (IQR 55.00-207.25) pairs, respectively. Temporal person-matched sampling required significantly fewer pairs than person-matched sampling (*P*<.01). Person-matched sampling and temporal person-matched sampling required significantly fewer samples than either random or temporal sampling (*P*<.001 for all pairwise comparisons). For these cohort-wide sampling methods, all 10,000 runs achieved significance within the 46,217 sampled pairs.

#### Within-Individual Sampling Methods

A subset of 8798, 9964, and 12,018 individuals from the dataset of 46,217 individuals did not reach significance in any of the 100 runs of within-individual, within-individual temporal, or within-individual sequential sampling, respectively. Thus, subsequent analyses of median and IQR include only the 30,462 individuals from each respective sampling method who reached significance in all 3 within-individual sampling methods. Of the individuals who did reach significance in at least 1 of 100 runs, they reached significance for a median of 7.0, 9.0, and 9.0 runs for within-individual, within-individual temporal, or within-individual sequential sampling, respectively (Multimedia Appendix 4). There was a weak negative Spearman correlation between the number of runs in which individuals failed to reach significance and the number of weeks they had available for sampling (Multimedia Appendix 5).

**Figure 2:**
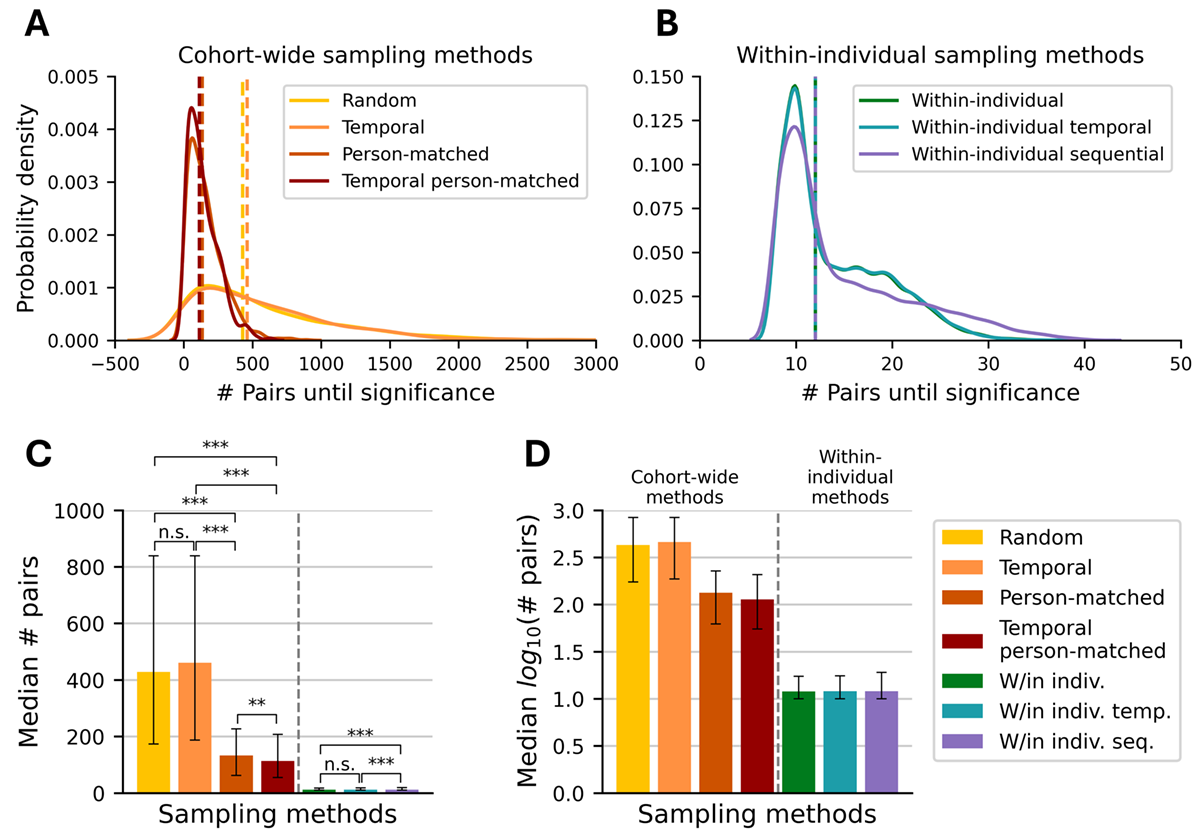
Comparison of the number of pairs of samples required for significance across all sampling methods. (A) Cohort-wide sampling methods. Dashed lines denote distribution medians. (B) Within-individual sampling methods; note the change in x-axis scale from (A) to (B). Dashed lines denote distribution medians. (C) Comparison of median pairs of samples required for significance with IQR error bars. (D) Rescaling of (C) on a logarithmic scale. n.s: nonsignificant; *P<.05; **P<.01; ***P<.001 using the Mann-Whitney U test for cohort-wide methods comparisons and the Wilcoxon Signed-Rank test for paired within-individual methods comparisons.

The median pairs of samples required to reach significance using within-individual sampling were 12.0 (IQR 10.00-17.50; Figures 2B-2D). Similarly, 12.0 (IQR 10.00-18.00) pairs of samples were required for within-individual temporal sampling. Within-individual sequential sampling, while also having the same median of 12.0 pairs of samples, was more variable with an IQR of 10.00-19.00 pairs of samples. Within-individual sequential sampling required a greater number of pairs of samples than both within-individual sampling and within-individual temporal sampling (*P*<.001). All within-individual sampling methods required significantly fewer samples to reach significance compared with any cohort-wide sampling method (*P*<.001 for all pairwise comparisons between cohort-wide and within-individual sampling methods).

### Effect Size at Significance

#### Cohort‑Wide Methods

**Figure 3:**
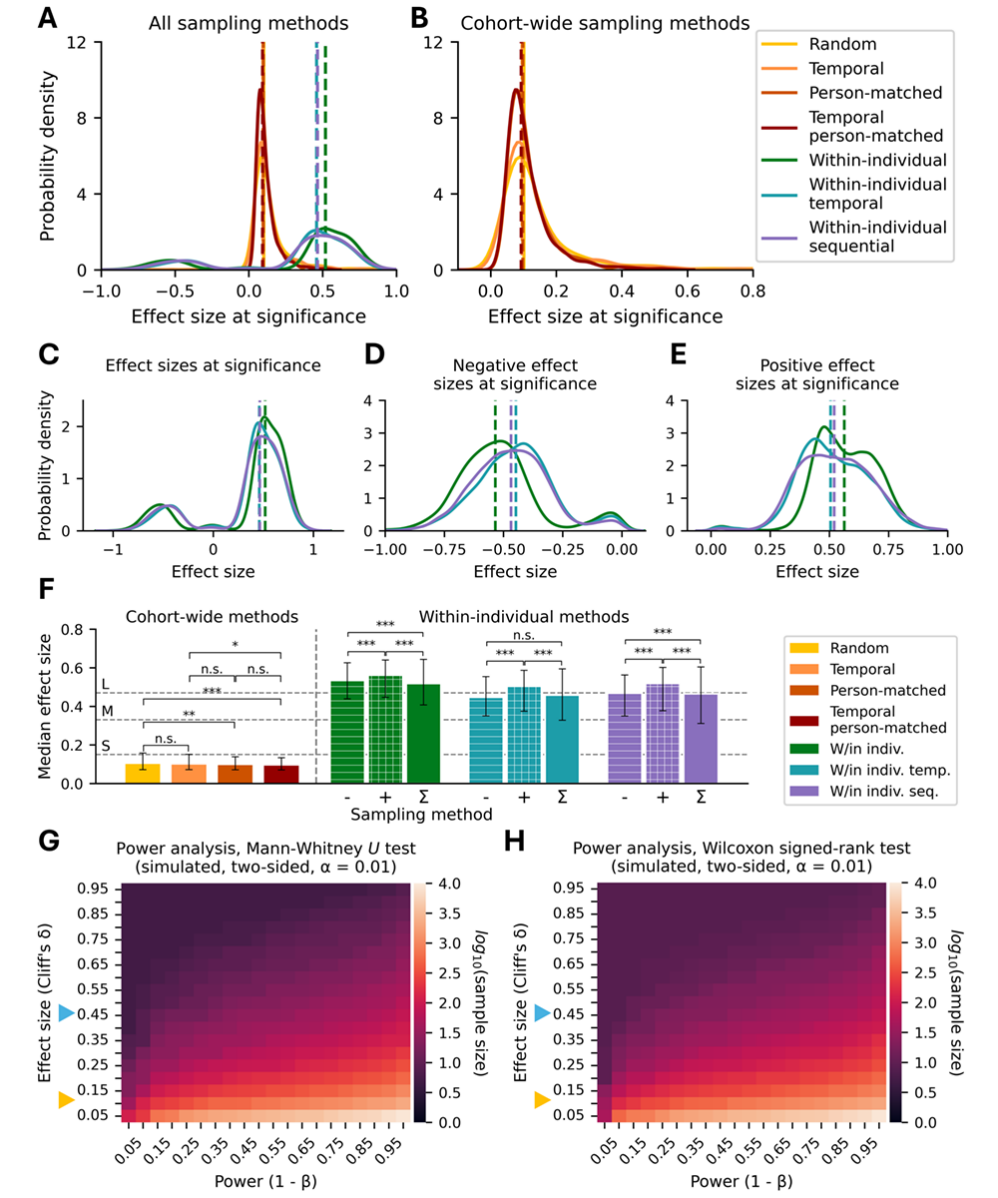
Effect size comparisons. (A) All sampling methods. (B) Cohort-wide sampling methods. (C) Within-individual sampling methods. (D) Stratified negative and (E) positive effect size distributions from (C). (F) Comparison of median effect size at significance for all sampling methods. (G) Empirical power analysis using the Mann-Whitney U test and (H) Wilcoxon Signed-Rank test at α=.01. Orange triangle: random sampling effect size. Blue triangle: approximate within-individual sampling methods effect size. n.s: non-significant; *P<.05; **P<.01; ***P<.001 using Mann-Whitney U test for cohort-wide and Kruskal-Wallis with the post hoc Dunn test for within-individual comparisons.

All cohort-wide methods had similar effect sizes: Random sampling required a median effect size of Cliff *δ*=0.10 (IQR 0.07-0.16; Figures 3A and 3B); the effect sizes for temporal sampling were comparable to random sampling at *δ*=0.10 (IQR 0.07-0.15). There was no significant difference in effect size between random and temporal sampling (*P*=.25). The median effect size of person-matched sampling was *δ*=0.10 (IQR 0.07-0.14). The median effect size of temporal person-matched sampling was *δ*=0.09 (IQR 0.07-0.13). There was no significant difference in effect size between person-matched and temporal person-matched sampling (*P*=.22).

#### Within-Individual Sampling Methods

All sampling methods that controlled for interindividual variability via within-individual sampling had effect sizes approximately 4-5 times that of cohort-wide sampling methods: The median effect size of within-individual sampling was *δ*=0.52 (IQR 0.41-0.64; Figures 3A and 3C). The median effect size of within-individual temporal sampling and within-individual sequential sampling was very similar at *δ*=0.46 (IQR 0.33-0.59) and *δ*=0.47 (IQR 0.31-0.60), respectively. There was a weak negative Spearman correlation between the number of runs in which individuals failed to reach significance and the effect size at significance (Multimedia Appendix 5). There was also a strong negative Spearman correlation between individuals’ effect size at significance and the number of weeks they have available for sampling (Multimedia Appendix 5).

We further stratified Figure 3C into the negative (Figure 3D) and positive (Figure 3E) effect sizes from which it was comprised. The median negative components of within-individual, within-individual temporal, and within-individual sequential sampling were *δ*=–0.53 (IQR –0.63 to –0.44), –0.45 (IQR –0.55 to –0.35), and –0.47 (IQR –0.56 to –0.35), respectively (Figure 3D); the positive components were *δ*=0.56 (IQR 0.45-0.64), 0.51 (IQR 0.37-0.59), and 0.52 (IQR 0.38-0.60), respectively (Figure 3E). When comparing the magnitude (ie, absolute value) of effect size among the negative, positive, and aggregated components of within-individual and within-individual sequential sampling, all effect sizes significantly differed from each other (*P*<.001; Figure 3F). For the positive (“+” in Figure 3F), negative (“–” in Figure 3F), and aggregated components of within-individual temporal sampling (“Σ” in Figure 3F), all effect sizes significantly differed from each other (*P*<.001) with the exception of the negative and aggregated component (*P*=.18).

We found that the magnitude of effect sizes at significance for cohort-wide sampling methods fell short of being categorized as “small” effects (Figure 3F; gray dashed lines) [25]. In contrast, all within-individual sampling methods either exceeded or were close to the threshold of “large” effects. Thus, all within-individual sampling methods had significantly greater effect size at significance compared with any cohort-wide sampling method (*P*<.001 for all pairwise comparisons between cohort-wide and within-individual sampling methods).

### Simulated Power Analysis

We compared the results from our study to those of a simulated power analysis (Figures 3G and 3H). Given our effect sizes at significance for within-individual sampling (*δ*=0.45 to 0.50), the aggregated sampling methods (*δ*=0.10) ought to require 26 times the number of samples to achieve a similar level of statistical power (80%). In practice, our aggregated random sampling method required 37 times the number of samples to achieve statistical significance relative to within-individual sampling methods.

## Discussion

### Principal Results

Our analyses found that longitude alone is enough to substantially reduce the number of comparisons needed to achieve a given level of significance. In addition, we found that within-individual comparisons across time allowed for the separation of multimodality—a second population could be identified with a different sign of effect than the aggregate or the majority population. This separation might not only be critical to the minority populations so identified, but the separation also led to increased effect sizes for each population—even the majority population benefited from the successful separation of these modes. By controlling for interindividual variability (that is, by comparing individuals to themselves across measurements), we reduced the number of samples required for statistical significance by as much as 40-fold compared with cross-sectional, cohort-wide methods. This capability afforded by longitudinal data is particularly critical in scenarios where large variability across individuals diminishes statistical power: the longitudinal measures allowed us to confidently separate individuals with different sign of responses (ie, those for whom weekends consistently raised HR vs those for whom it significantly lowered HR), so that power and representation of both negative and positive weekend effects were both improved by virtue of the longitudinal measures. This is in contrast to cohort-wide sampling methods, where effect size values were 4-5 times smaller than those found in within-individual sampling, and overwhelmingly positive. Said differently, cross-sectional analyses led the smaller group of individuals with negative weekend effects on HR to be effectively snowed under by the larger group with weekend HR rise, and the individual-level response heterogeneity was lost. This separation of subpopulations by consistent effect size would not be possible without longitudinal resampling to reveal consistency of responses to weekends by individual. Once the population was separated into those with positive and those with negative weekend HR change, we found that the cross-sectional population effect size was significantly underrepresenting the actual effect size of the subpopulation with positive change on weekends; even the majority population that was not snowed under was not represented faithfully by the cross-sectional analysis. By leveraging the within-individual measurements across time afforded by wearables, this work demonstrates that longitudinal data can simultaneously enhance statistical power and reveal response heterogeneity in real-world applications.

It is worth noting that the most commonly used statistical comparisons assume all data points to be independent, whereby repeated measures of an individual would be considered pseudoreplication. Indeed, in previous publications, we have encountered reviewers objecting that time-series analysis using within-individual comparisons is never statistically valid for this reason. To ignore the possible gains enabled by longitudinal data now becoming common would be a costly mistake. Lack of independence should not be confused with the value of longitudinal, repeated measures of real-world responses—here, HR on weekends and weekdays as a model system in which to test these comparisons. What longitude allows for is the construction of within-individual “baseline” representations of HR against which deviations from that baseline can be measured repeatedly to test for consistency of response per individual. To account for this potential confusion, we reduced our cross-sectional comparisons to a single response per individual. This removed pseudoreplication from the cross-sectional model. However, we assumed the responses of each individual to the same event should not be random, but that instead individuals might consistently be of a phenotype (as in, responder or nonresponder to some drug in a clinical trial). Leveraging longitude to measure consistency per individual and getting away from the concern about pseudoreplication allowed us to test this hypothesis and find substantial support for it. Longitudinal resampling revealed that people fell into definite types (ie, signs of HR response to weekends). Such approaches might therefore be leveraged in clinical trials to assess consistency within individuals of response to treatment events. This, in turn, might allow deeper future analysis to predict responders and non-responders to treatments. It is interesting to note that sequential and temporal within-individual sampling did not amplify the effect of within-individual longitude alone, suggesting that such future trials might not need to be overly constrained by pairing measurements in time, but liberated instead to simply amass multiple comparisons from which to determine which “phenotype” an individual best fits.

Interestingly, there were a few individuals with neutral (ie, *δ*=0) weekend effects. Most individuals exhibited increases in HR during weekends, while a sizable minority exhibited decreases in HR. Though not the main focus of this work, to our knowledge, this is the first report of weekend effects being so persistent across such a majority of individuals. Behaviors commonly associated with weekends (eg, increased social excursions, greater alcohol intake) may help explain why nighttime HR changes differently for some individuals on these days compared with weekdays. In addition, these changes in HR (and underlying behaviors that drive them) may be associated with differential health outcomes. Further research is required to determine the relationship between weekend habits, HR, and long-term health outcomes.

### Limitations

This work has several limitations to which we would draw attention. We assessed HR, which is highly variable, and not all physiological outputs exhibit the same variability, so validations should be conducted for other modalities. We used typical Cliff’s *δ* cutoffs to categorize “small,” “medium,” and “large” effects. However, these are artificial categories, and effect sizes ought to be determined by experts in specific conditions when applying this approach to different outcomes. Other modalities than resting HR could be expected to show different patterns of change on weekends, depending on the behaviors reflected by each modality (eg, glucose or temperature might show different kinds of weekend effects than HR). These would be important explorations when describing the health impacts of weekend-associated social jet lag.

There are a few socioeconomic caveats to these analyses. First is our assumption that Friday and Saturday nights constitute each individual’s weekend nights. Approximately 32.5% of Americans typically work on Saturdays, Sundays, or holidays, which may lower the incidence and weaken our assumption of weekend-weekday night delineation for individuals across different populations [26]. We did not attempt to correct for this, as it did not seem to affect our analyses significantly, the point of which was to compare various statistical approaches to leveraging longitudinal data, and not to precisely describe the phenomenology of weekends on health. Second, our study cohort was composed overwhelmingly of White individuals residing in the northern hemisphere. While this methodology of within-individual sampling can be applied to longitudinal data for any individual, it should not be taken for granted that similar magnitudes of change in HR (or other modalities) will be found among study cohorts with different demographic compositions.

We note that a sizeable proportion of our study cohort (ie, 19% to 26% depending on sampling method) never reached statistical significance in a given within-individual sampling method. This observation is likely due to (1) some individuals having less data from which to sample, and (2) smaller effects of weekends being more difficult to detect using statistical tests. In the case of individuals with less data, large and consistent changes in weekend HR would be required to reach statistical significance when fewer observations are available. In contrast, individuals with more data but smaller changes in weekend HR may have failed to reach significance due to the effect of weekends on HR being too weak to detect; that is, comparisons should correctly fail to find significant differences in individuals for whom weekends are not causing change, which here was found to be about one-fifth of the population. In both cases, having more longitudinally sampled data can increase statistical power for detecting effects of all magnitudes. In addition, having more data can help clarify the magnitude and direction of change in HR, which becomes essential to report as greater sample sizes with high statistical power will frequently reach statistical significance.

### Conclusions

Our results support study designs using longitudinal, within-individual measures when looking for biological or behavioral changes within heterogeneous populations. As the COVID-19 pandemic has helped reveal, having precise, within-individual physiological baselines is critical to identifying states of abnormal physiology on a personalized level [13,19,27,28]. As algorithms based on within-individual, longitudinal comparisons become more common in applied settings, we hope analyses like those we provided here will expand the adoption of methods to control for nonrandom sources of variance. By using within-individual measures, we efficiently identified heterogeneous, individual-level physiological responses to weekends that were indistinguishable using traditional aggregation and random sampling methods. Our methods highlighted not only the benefits of within-individual measures afforded by wearable devices but also the large physiological heterogeneity between individuals that such longitudinal measures can reveal.

## Appendix

Multimedia Appendix 1 Illustration of a few example iterations of each sampling method.

Multimedia Appendix 2 Mean and SD for each sampling method at *P*<.01

Multimedia Appendix 3 Median, IQR, mean, and SD for each sampling method at *P*<.05.

Multimedia Appendix 4 Median number of runs in which individuals did not reach significance by sampling method.

Multimedia Appendix 5 Correlations between effect size, reaching statistical significance, and data availability.

## Abbreviations

HR: heart rate
IID: independent and identically distributed

## Data Availability

Oura’s data use policy does not permit us to make wearable device data (collected via the Oura Ring) available to third parties. Access to anonymized and privacy-protected data may be granted to a qualified academic investigator upon completing agreements with Oura Health Oy and the investigators. Please contact Ashley E. Mason and Benjamin L. Smarr to obtain an application to obtain these data.
