# Supplementary material for: Testing the Impact of Intensive, Longitudinal Sampling on Assessments of Statistical Power and Effect Size Within a Heterogeneous Human Population: Natural Experiment Using Change in Heart Rate on Weekends as a Surrogate Intervention
Source: J Med Internet Res. 2025 May 21;27:e60284. doi: 10.2196/60284 (PMC12138295; doi:10.2196/60284)
Supplement: Multimedia Appendix 1 [file jmir_v27i1e60284_app1.pdf]

The Baseline: Random Sampling

Key characteristics:

- Accounts for **neither time- nor person-dependent** differences in HR.
- Is the shuffled version of Temporal Person-Matched Sampling (i.e., both person-matching and time-matching are disrupted).

Random Sampling

	Weekdays					Weekends	
1	74.42	62.90	58.30	66.50	64.10	69.41	69.70
2	53.05	53.36	57.88	52.22	54.77	56.75	61.55
⋮	⋮	⋮	⋮	⋮	⋮	⋮	⋮
46,216	77.57	63.15	69.80	71.22	88.24	76.77	76.49
46,217	75.68	75.15	73.91	77.06	72.68	74.25	74.35
	Weekdays					Weekends	

Random Sampling

Data can be sampled from any individual (without replacement) from any week

Weekdays

$p = 1.00$

$\delta = 1.00$

Weekends

Individual	Weekdays					Weekends	
1	74.42	62.90	58.30	66.50	64.10	69.41	69.70
2	**53.05**	53.36	57.88	52.22	54.77	56.75	61.55
⋮	⋮	⋮	⋮	⋮	⋮	⋮	⋮
46,216	77.57	63.15	69.80	71.22	88.24	76.77	76.49
46,217	75.68	75.15	73.91	77.06	72.68	74.25	**74.35**

Random Sampling

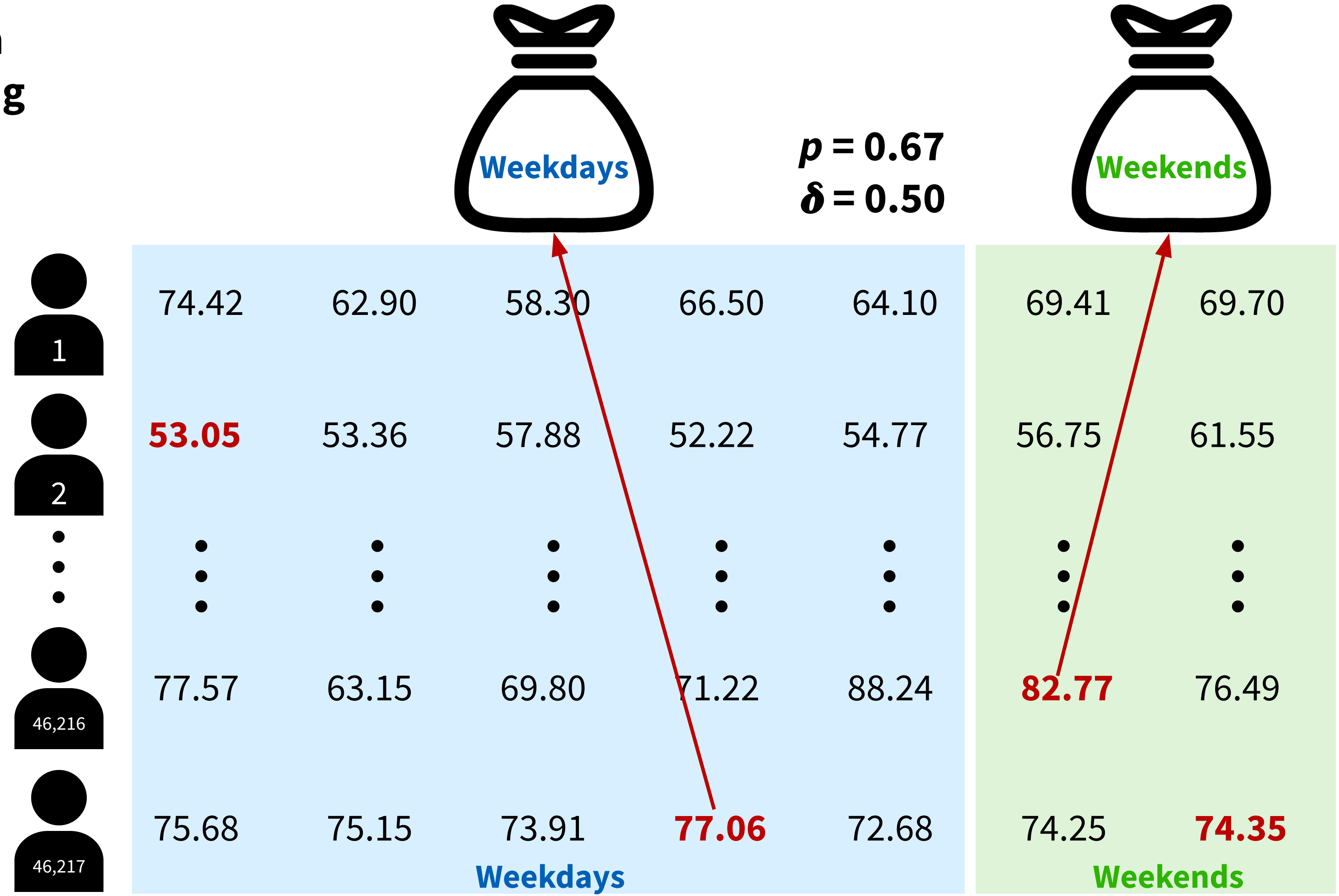

Random Sampling

$p = 0.70$
$\delta = 0.33$

	Weekdays					Weekends	
1	74.42	62.90	58.30	66.50	64.10	69.41	**69.70**
2	**53.05**	53.36	57.88	52.22	54.77	56.75	61.55
⋮	⋮	⋮	⋮	⋮	⋮	⋮	⋮
46,216	77.57	63.15	**69.80**	71.22	88.24	**76.77**	76.49
46,217	75.68	75.15	73.91	**77.06**	72.68	74.25	**74.35**

Random Sampling

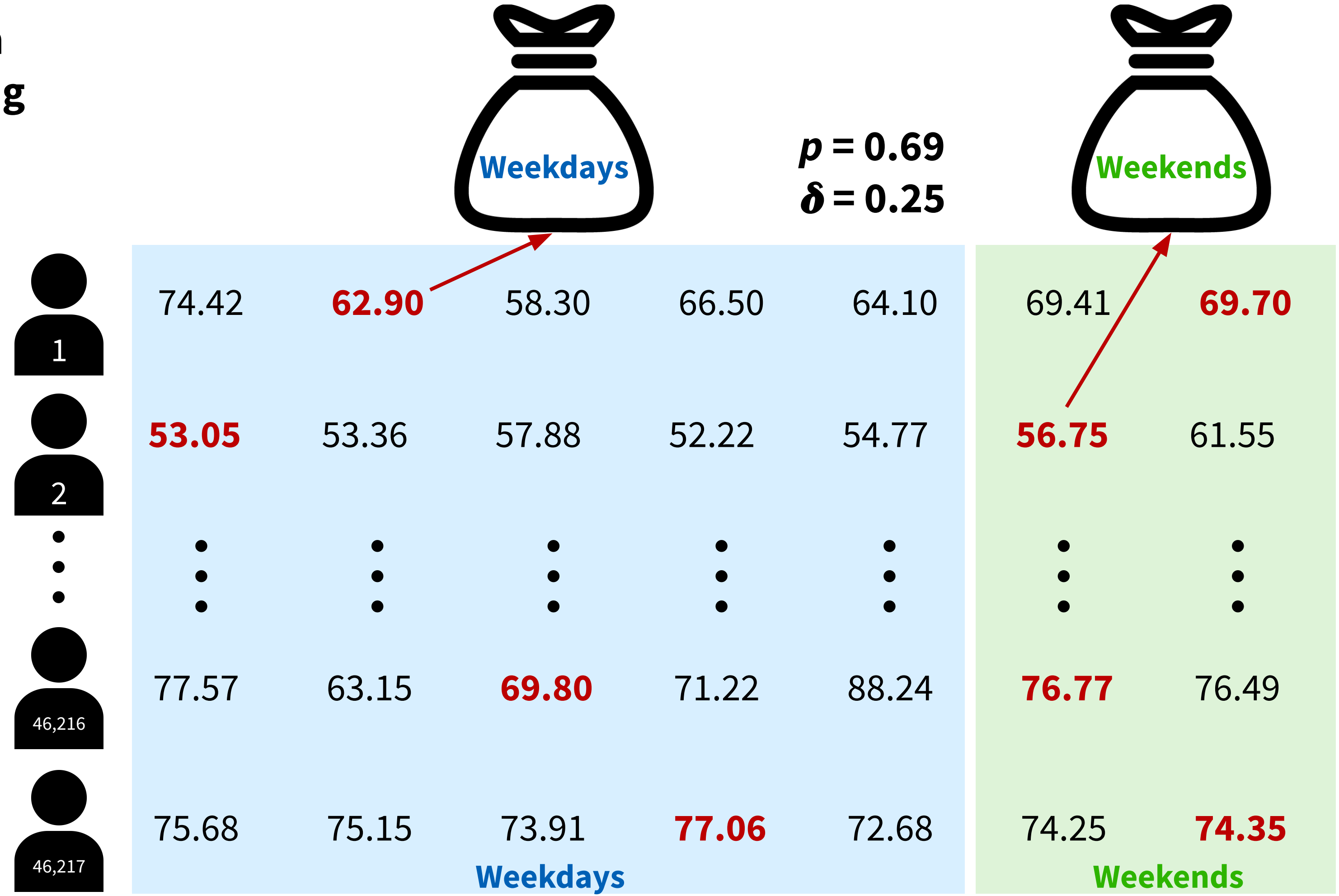

Temporal Sampling

Key characteristics:

- Accounts for only **time-dependent** differences in HR.
- Is the shuffled version of Temporal Person-Matched Sampling, if we shuffle within-weeks (i.e., HR data is shuffled among calendar week *n* such that week-matching is maintained, but the data is no longer person-matched).

Temporal Sampling

Randomly chosen week: 33

Weekdays | Weekends

	Weekdays					Weekends	
1	68.87	60.57	68.08	58.31	60.35	67.65	62.74
2	54.34	52.79	52.63	55.25	55.78	54.63	56.61
⋮	⋮	⋮	⋮	⋮	⋮	⋮	⋮
46,216	68.53	67.44	68.62	71.07	79.88	67.61	75.90
46,217	75.50	71.96	75.55	74.14	71.66	73.40	76.91

Temporal Sampling

Randomly chosen week: 33

$p = 1.00$
$\delta = -1.0$

	Weekdays					Weekends	
1	68.87	60.57	68.08	58.31	60.35	67.65	62.74
2	54.34	52.79	52.63	55.25	55.78	54.63	**56.61**
⋮	⋮	⋮	⋮	⋮	⋮	⋮	⋮
46,216	68.53	67.44	68.62	71.07	79.88	67.61	75.90
46,217	75.50	71.96	75.55	74.14	**71.66**	73.40	76.91

Weekdays

Weekends

Temporal Sampling

Randomly chosen week: 9

***p* = 1.00**
***δ* = -0.50**

User	Weekdays					Weekends	
1	69.21	66.99	74.59	58.44	56.17	**66.74**	65.49
2	**58.07**	59.36	50.98	55.31	54.07	56.81	58.21
⋮	⋮	⋮	⋮	⋮	⋮	⋮	⋮
46,216	69.47	63.75	72.45	70.53	68.55	78.26	72.17
46,217	73.09	72.69	71.36	77.64	72.99	74.38	72.64

Weekdays → Weekdays bag: 58.07

Weekends → Weekends bag: 66.74

Temporal Sampling

Randomly chosen week: 23

p **= 0.75**

δ = -0.33

	Weekdays					Weekends	
1	74.42	62.90	58.30	66.50	64.10	69.41	69.70
2	53.05	53.36	57.88	52.22	54.77	56.75	61.55
⋮	⋮	⋮	⋮	⋮	⋮	⋮	⋮
46,216	**77.57**	63.15	69.56	71.22	88.24	76.77	76.49
46,217	75.68	75.15	73.91	77.06	72.68	74.25	**74.35**

Temporal Sampling

Randomly chosen week: 14

$p = 0.88$

$\delta = 0.00$

	Weekdays					Weekends	
1	72.58	61.66	**59.56**	68.60	65.10	73.70	70.30
2	52.16	49.84	55.68	51.97	52.67	57.83	63.02
⋮	⋮	⋮	⋮	⋮	⋮	⋮	⋮
46,216	70.21	62.41	66.05	69.56	74.26	**74.95**	75.76
46,217	76.04	75.45	72.85	76.38	72.27	73.85	76.94

Weekdays → Weekdays bag: 59.56

Weekends → Weekends bag: 74.95

Person-Matched

Key characteristics:

- Accounts for only **person-dependent** differences in HR.
- Is the version of Temporal Person-Matched Sampling if instead of choosing the weekend-weekday HR pair from the individual's same week of data, weekend HR is chosen from a random week, and weekday HR is also chosen from a random week (i.e., person-matching is maintained, but the data is no longer time-matched).

Person-Matched Sampling

Data from each individual can be from any week

Person	Weekdays					Weekends	
1	74.42	62.90	58.30	66.50	64.10	69.41	69.70
2	53.05	53.36	57.88	52.22	54.77	56.75	61.55
⋮	⋮	⋮	⋮	⋮	⋮	⋮	⋮
46,216	77.57	63.15	69.56	71.22	88.24	76.77	76.49
46,217	75.68	75.15	73.91	77.06	72.68	74.25	74.35
	Weekdays (from any week)					Weekends (from any week)	

Person-Matched Sampling

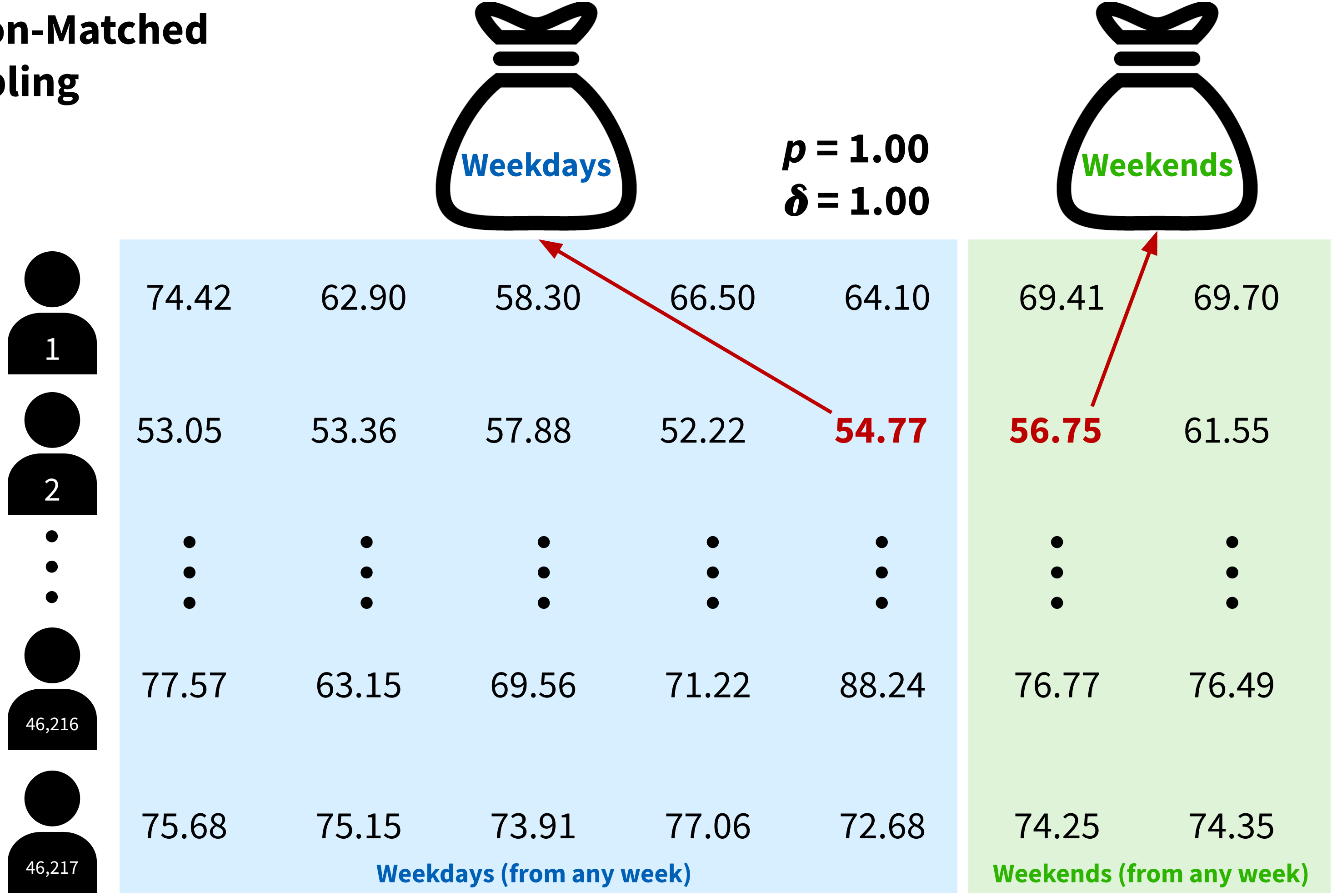

Person-Matched Sampling

$p = 0.50$
$\delta = 0.50$

Weekdays | Weekends

Person	Weekdays (from any week)					Weekends (from any week)	
1	74.42	**62.90**	58.30	66.50	64.10	**69.41**	69.70
2	53.05	53.36	57.88	52.22	54.77	56.75	61.55
⋮	⋮	⋮	⋮	⋮	⋮	⋮	⋮
46,216	77.57	63.15	69.56	71.22	88.24	76.77	76.49
46,217	75.68	75.15	73.91	77.06	72.68	74.25	74.35

Person-Matched Sampling

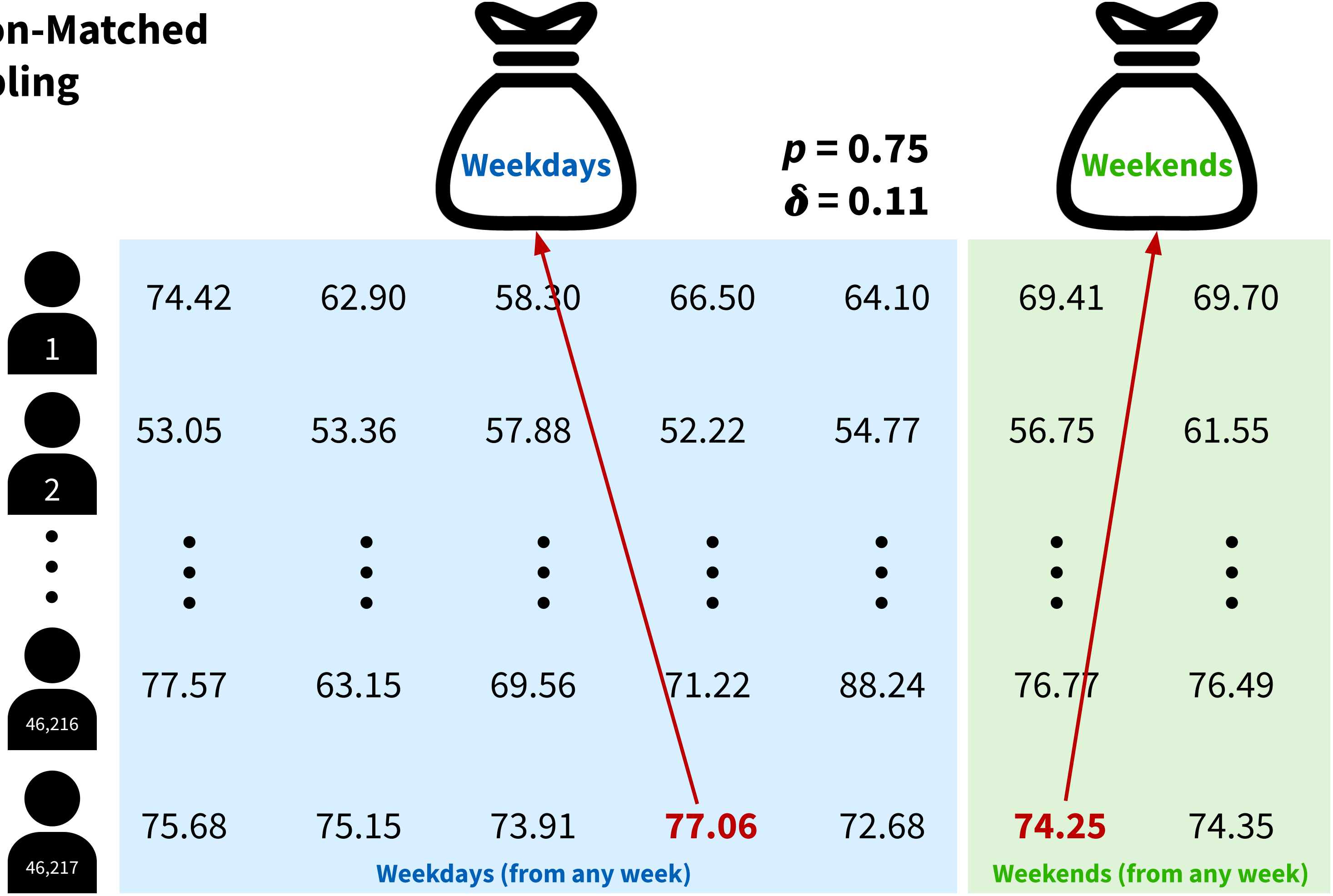

Person-Matched Sampling

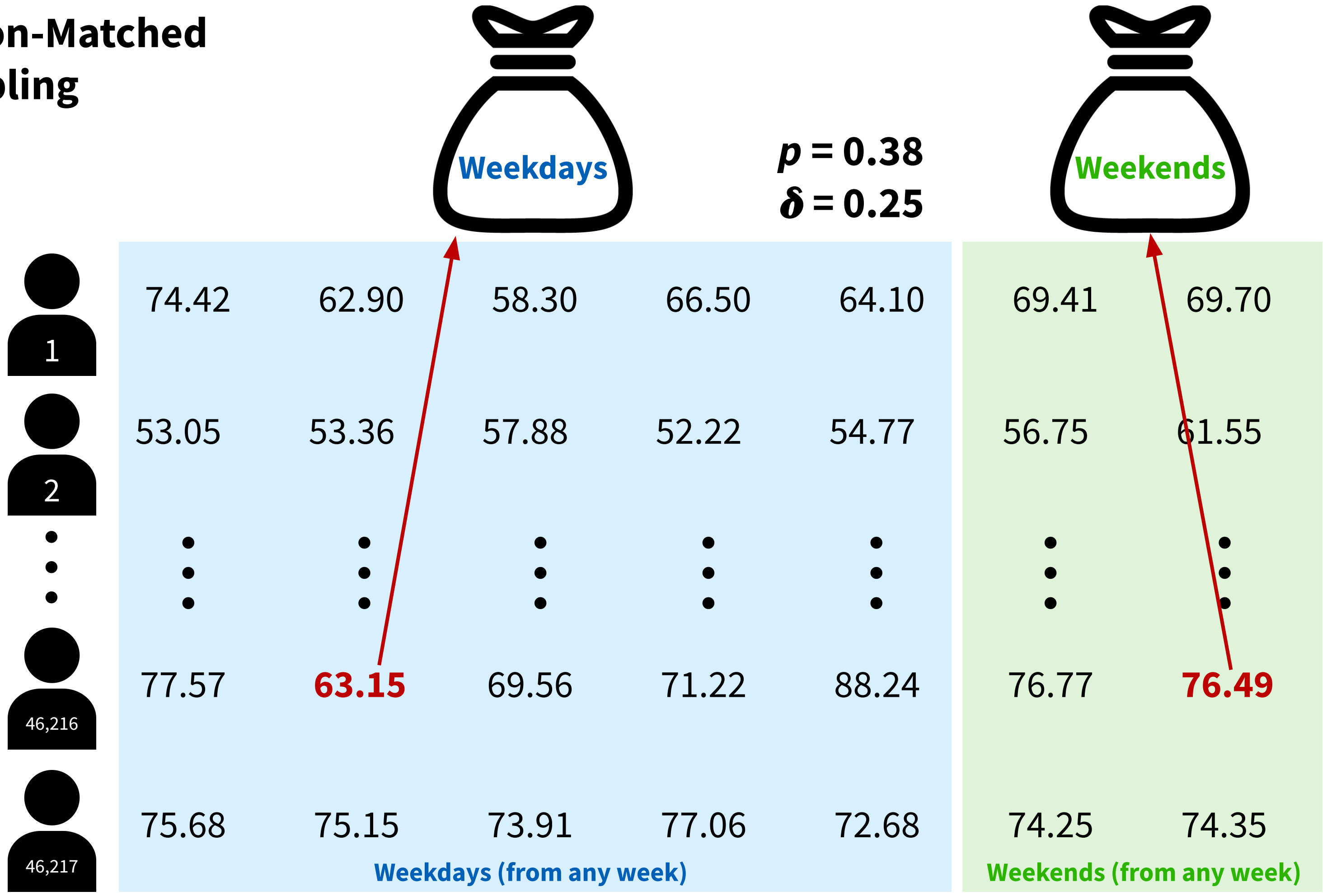

Temporal Person-Matched Sampling

Key characteristics:

- Accounts for both **person-dependent** and **time-dependent** differences in HR.
- One weekend-weekday sample of HR is chosen from each individual in the dataset, and the weekend and weekday HR values are from the same calendar week.

Temporal Person-Matched Sampling

Randomly chosen week: 33

Person	Weekdays					Weekends	
1	68.87	60.57	68.08	58.31	60.35	67.65	62.74
2	54.34	52.79	52.63	55.25	55.78	54.63	56.61
⋮	⋮	⋮	⋮	⋮	⋮	⋮	⋮
46,216	68.53	67.44	68.62	71.07	79.88	67.61	75.90
46,217	75.50	71.96	75.55	74.14	71.66	73.40	76.91
	Weekdays					Weekends	

Temporal Person-Matched Sampling

Randomly chosen week: 33

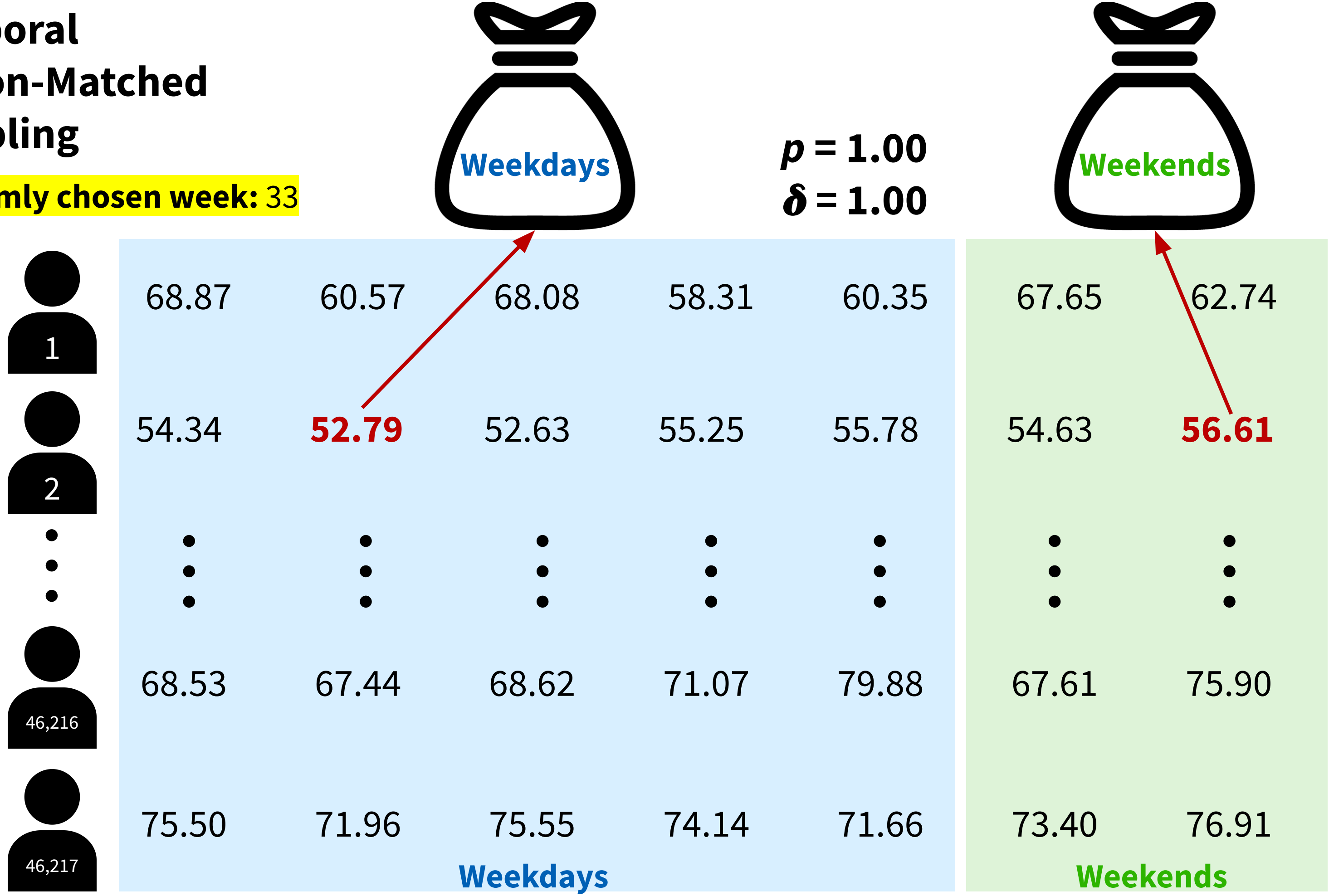

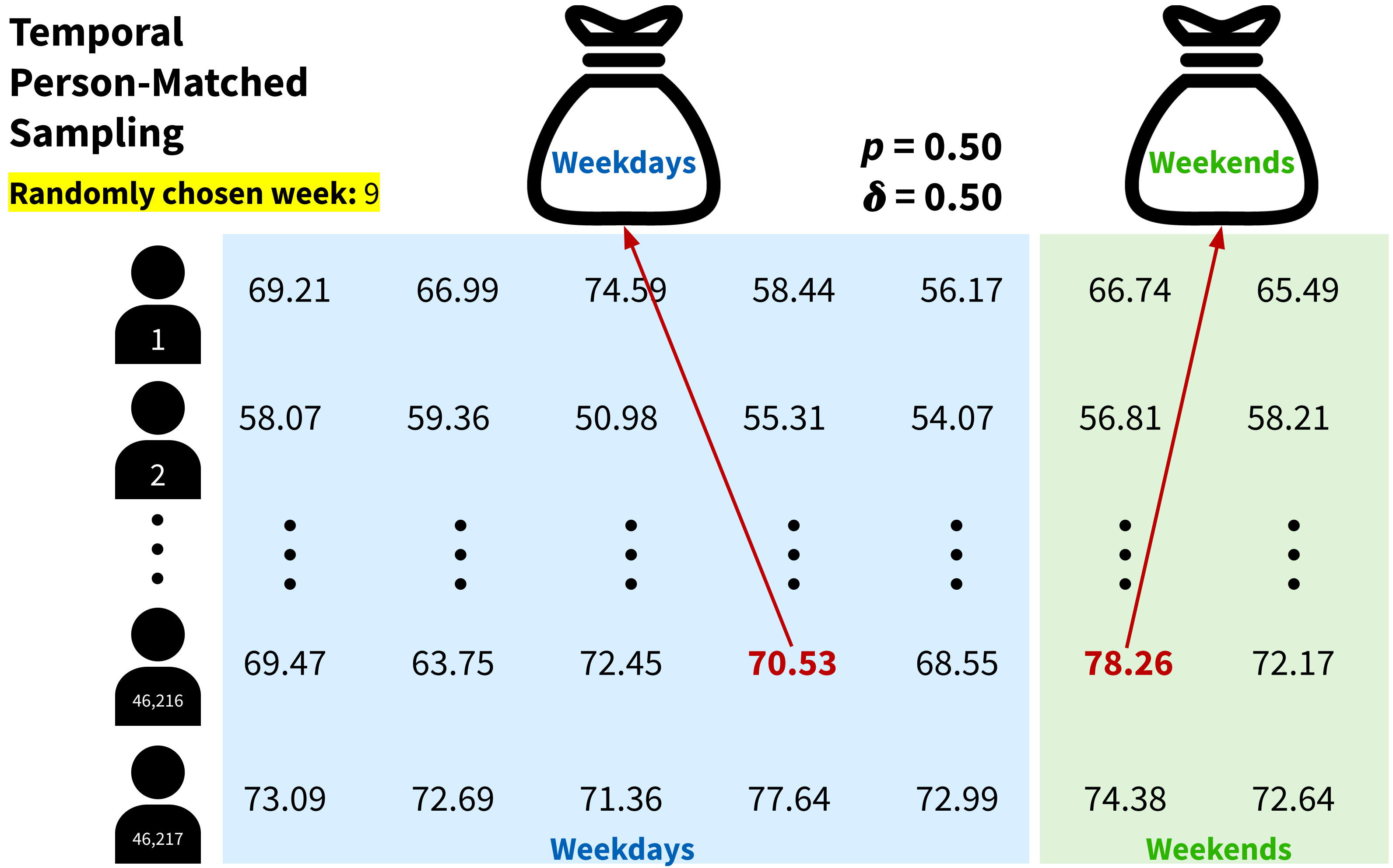

Temporal Person-Matched Sampling
Randomly chosen week: 9
Weekdays
p = 0.50
δ = 0.50
Weekends
1
2
46,216
46,217
69.21 66.99 74.59 58.44 56.17
58.07 59.36 50.98 55.31 54.07
69.47 63.75 72.45 70.53 68.55
73.09 72.69 71.36 77.64 72.99
66.74 65.49
56.81 58.21
78.26 72.17
74.38 72.64
Weekdays
Weekends

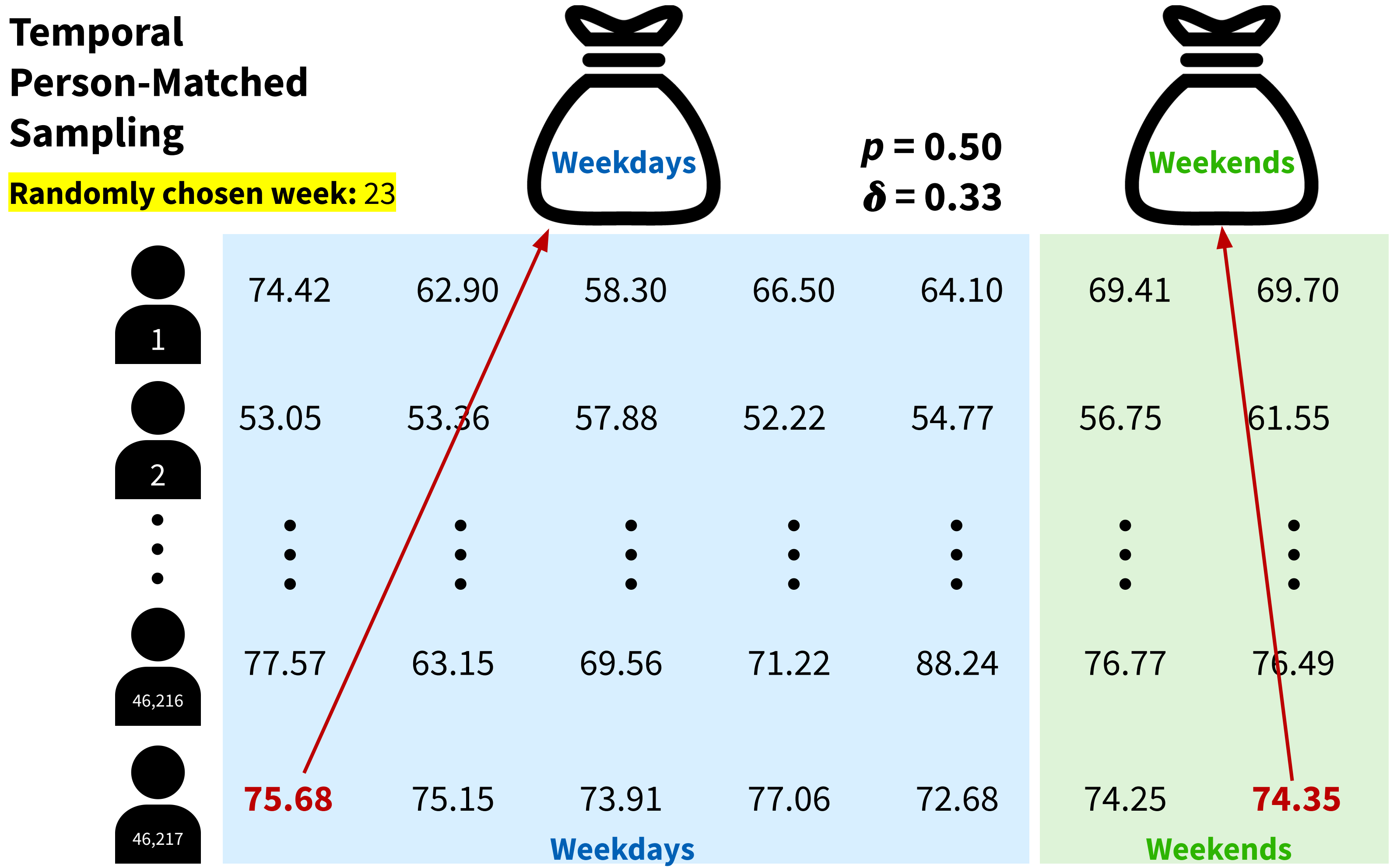
Temporal
Person-Matched
Sampling
Randomly chosen week: 23
Weekdays
Weekends
p = 0.50
δ = 0.33
1
74.42 62.90 58.30 66.50 64.10
69.41 69.70
2
53.05 53.36 57.88 52.22 54.77
56.75 61.55
46,216
77.57 63.15 69.56 71.22 88.24
76.77 76.49
46,217
75.68 75.15 73.91 77.06 72.68
74.25 74.35
Weekdays
Weekends

Temporal Person-Matched Sampling

Randomly chosen week: 14

$p = 0.25$
$\delta = 0.38$

Person	Weekdays					Weekends	
1	72.58	61.66	59.56	68.60	**65.10**	**73.70**	70.30
2	52.16	49.84	55.68	51.97	52.67	57.83	63.02
⋮	⋮	⋮	⋮	⋮	⋮	⋮	⋮
46,216	70.21	62.41	66.05	69.56	74.26	74.95	75.76
46,217	76.04	75.45	72.85	76.38	72.27	73.85	76.94

Within-Individual Sampling

Key characteristics:

- Weekend-weekday sampling done within-person entirely.
- Accounts for both **person-dependent** differences in HR.
- Pairs of weekend-weekday HR are chosen randomly from each individual's data, and the weekend and weekday HR values may be from any calendar week.

Within-Individual Sampling

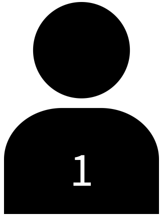

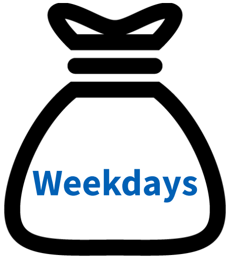

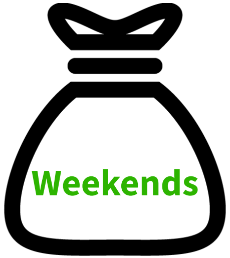

Week 1	65.21	66.45	66.21	64.80	67.62	67.20	67.15
Week 2	66.17	63.95	67.16	67.94	63.38	69.06	67.03
⋮	⋮	⋮	⋮	⋮	⋮	⋮	⋮
Week 45	64.65	66.02	65.60	66.45	62.25	64.19	68.56
Week 46	65.47	62.54	67.99	62.89	65.49	65.47	63.55
	Weekdays (from any week)					Weekends (from any week)	

Within-Individual Sampling

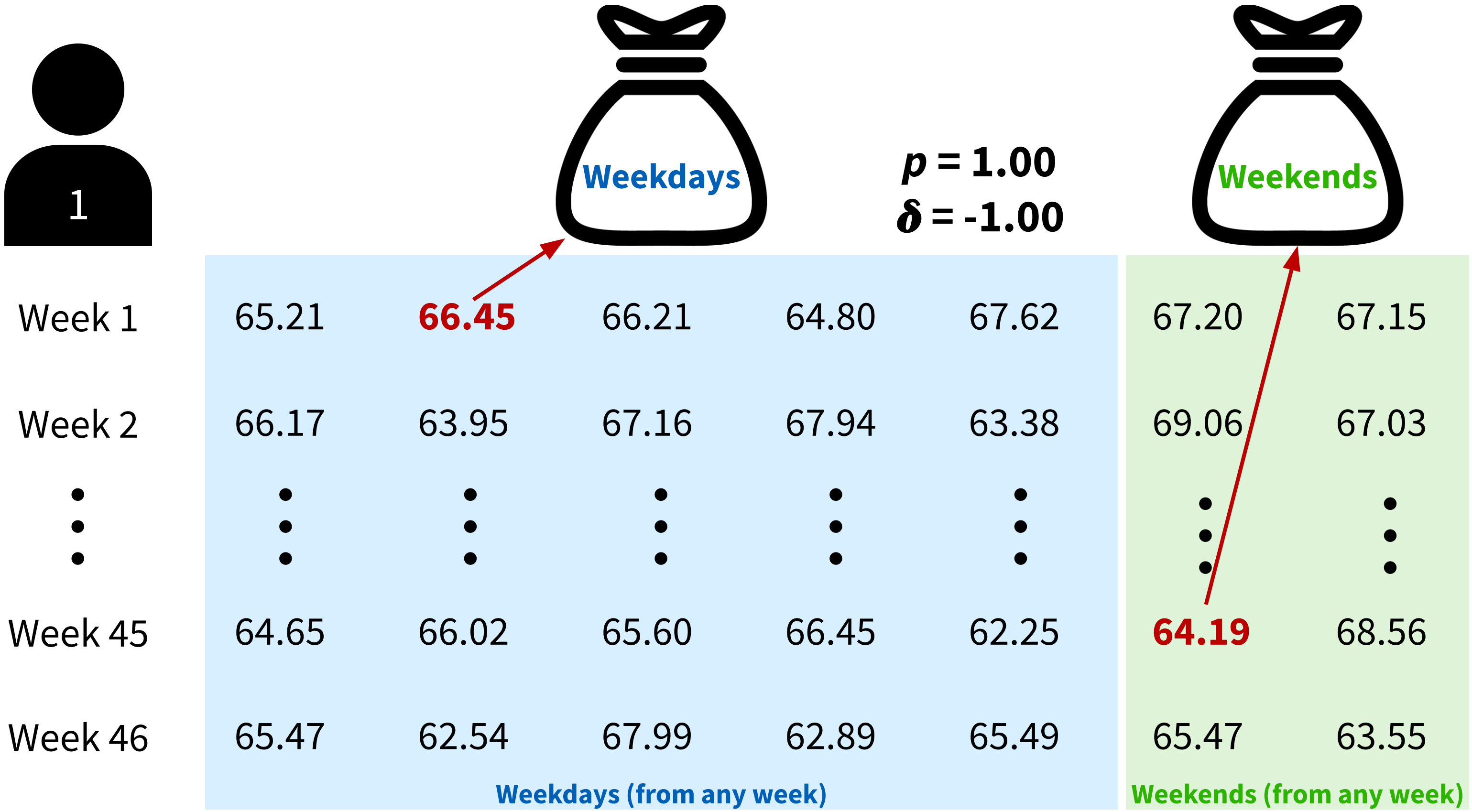

Within-Individual Sampling

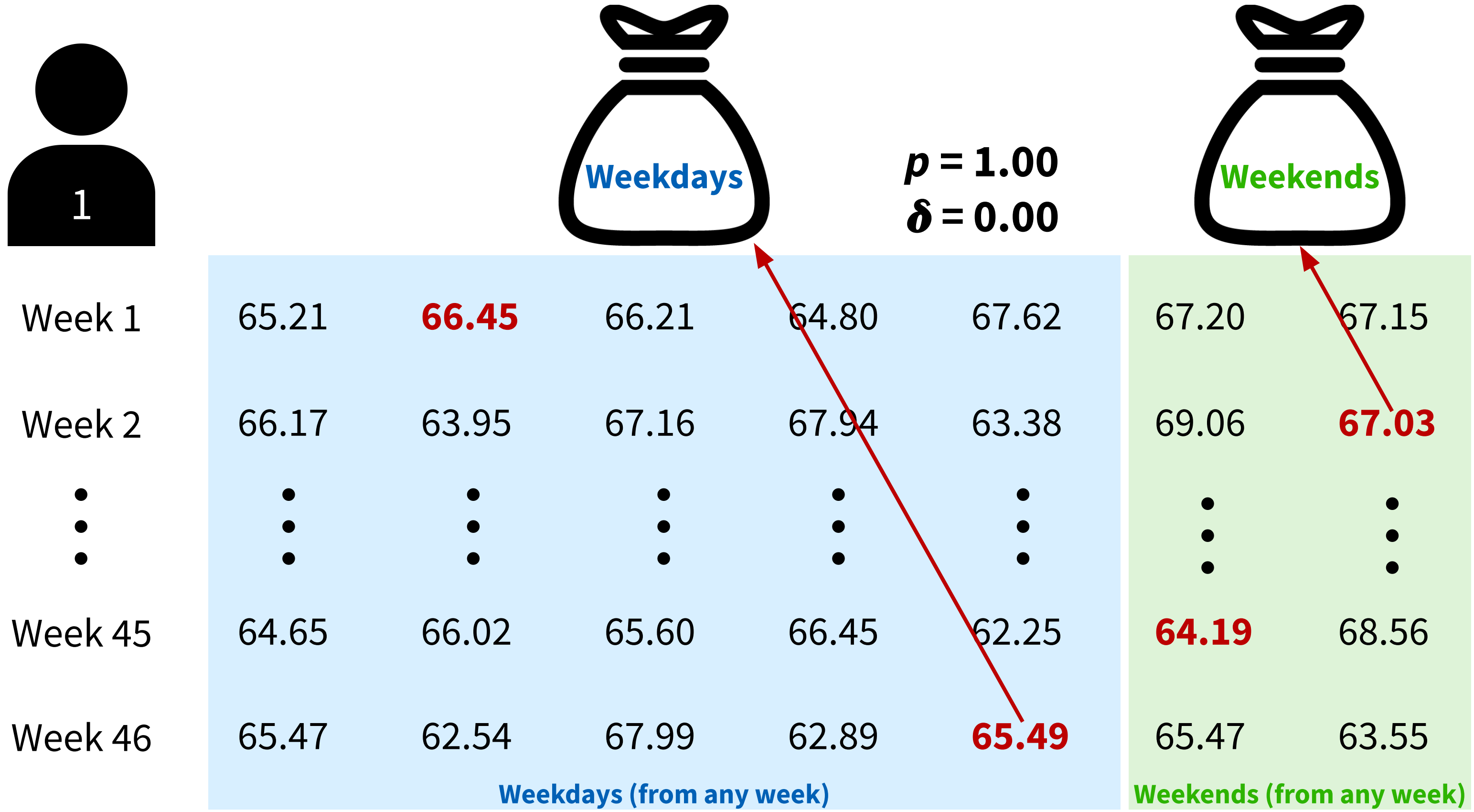

Within-Individual Sampling

1

Weekdays

$p = 0.75$
$\delta = 0.56$

Weekends

Week 1	65.21	**66.45**	66.21	64.80	67.62	67.20	67.15
Week 2	66.17	63.95	67.16	67.94	63.38	**69.06**	**67.03**
⋮	⋮	⋮	⋮	⋮	⋮	⋮	⋮
Week 45	64.65	66.02	65.60	66.45	**62.25**	**64.19**	68.56
Week 46	65.47	62.54	67.99	62.89	**65.49**	65.47	63.55
	Weekdays (from any week)					Weekends (from any week)	

Within-Individual Sampling

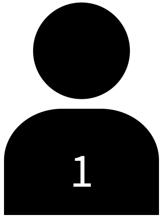

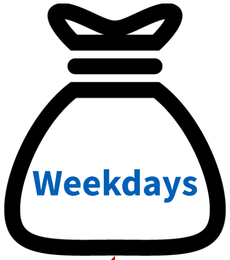

$p = 0.38$
$\delta = 0.75$

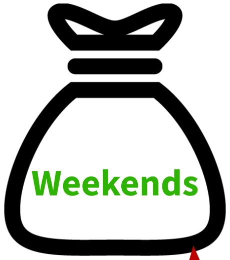

	Weekdays (from any week)					Weekends (from any week)	
Week 1	65.21	**66.45**	66.21	64.80	67.62	67.20	**67.15**
Week 2	66.17	63.95	67.16	67.94	63.38	**69.06**	**67.03**
⋮	⋮	⋮	⋮	⋮	⋮	⋮	⋮
Week 45	64.65	66.02	65.60	66.45	**62.25**	**64.19**	68.56
Week 46	65.47	**62.54**	67.99	62.89	**65.49**	65.47	63.55

Within-Individual Temporal Sampling

Key characteristics:

- Weekend-weekday sampling done within-person entirely.
- Accounts for both **person-dependent** and **time-dependent** differences in HR.
- Pairs of weekend-weekday HR are randomly chosen from each individual’s data, and the weekend and weekday HR values are from the same random calendar week.

Within-Individual Temporal Sampling

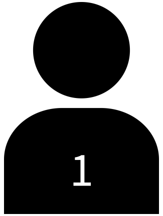

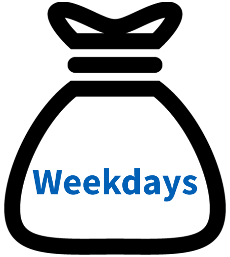

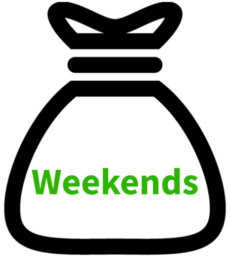

Week 1	65.21	66.45	66.21	64.80	67.62	67.20	67.15
Week 2	66.17	63.95	67.16	67.94	63.38	69.06	67.03
⋮	⋮	⋮	⋮	⋮	⋮	⋮	⋮
Week 45	64.65	66.02	65.60	66.45	62.25	64.19	68.56
Week 46	65.47	62.54	67.99	62.89	65.49	65.47	63.55
			Weekdays			Weekends	

Within-Individual Temporal Sampling

1

$p = 1.00$
$\delta = 1.00$

	Weekdays					Weekends	
Week 1	65.21	66.45	**66.21**	64.80	67.62	**67.20**	67.15
Week 2	66.17	63.95	67.16	67.94	63.38	69.06	67.03
⋮	⋮	⋮	⋮	⋮	⋮	⋮	⋮
Week 45	64.65	66.02	65.60	66.45	62.25	64.19	68.56
Week 46	65.47	62.54	67.99	62.89	65.49	65.47	63.55

Within-Individual Temporal Sampling

1

Weekdays

p = 0.50
δ = 0.50

Weekends

	Weekdays					Weekends	
Week 1	65.21	66.45	**66.21**	64.80	67.62	**67.20**	67.15
Week 2	66.17	63.95	67.16	**67.94**	63.38	**69.06**	67.03
⋮	⋮	⋮	⋮	⋮	⋮	⋮	⋮
Week 45	64.65	66.02	65.60	66.45	62.25	64.19	68.56
Week 46	65.47	62.54	67.99	62.89	65.49	65.47	63.55

Within-Individual Temporal Sampling

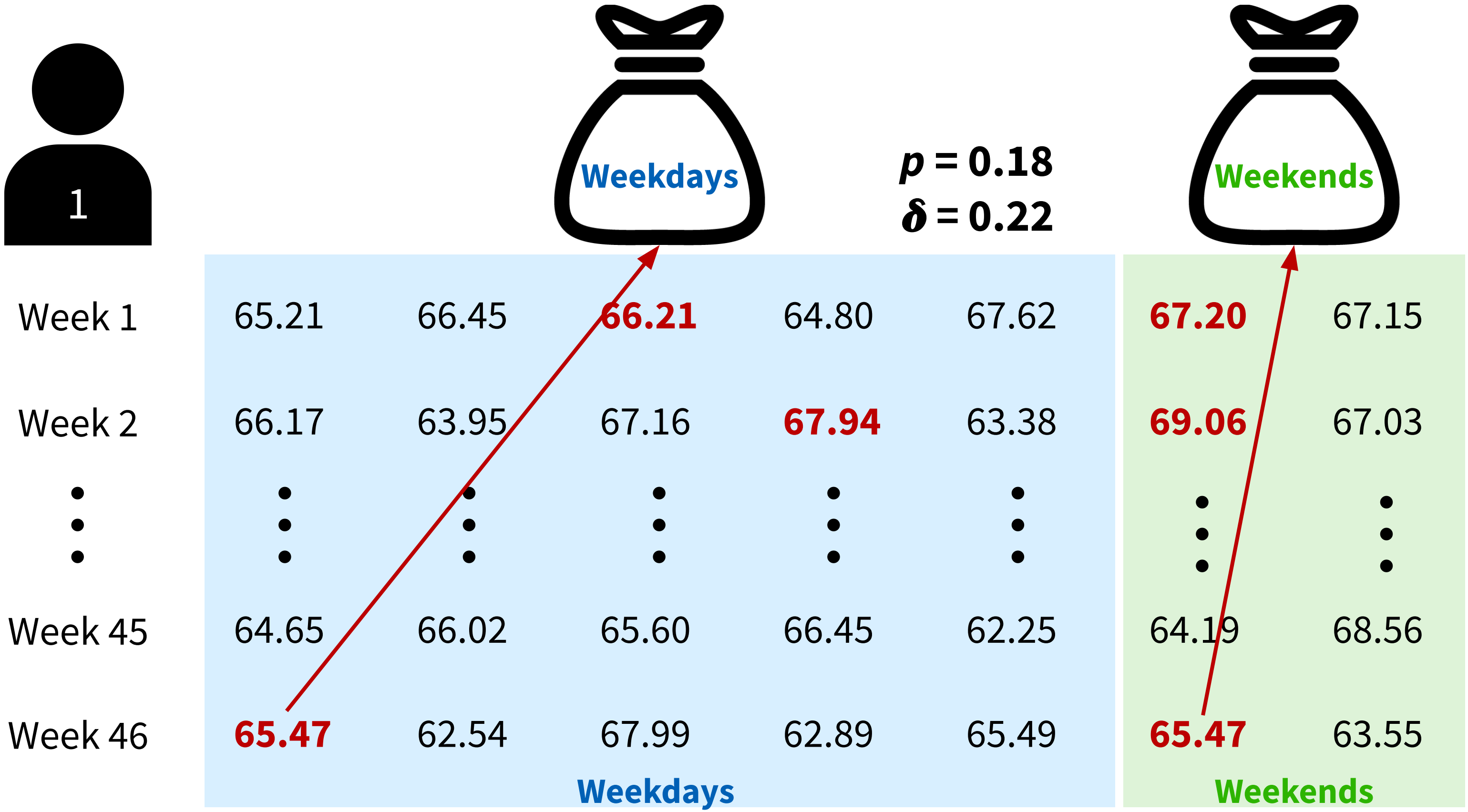

Within-Individual Temporal Sampling

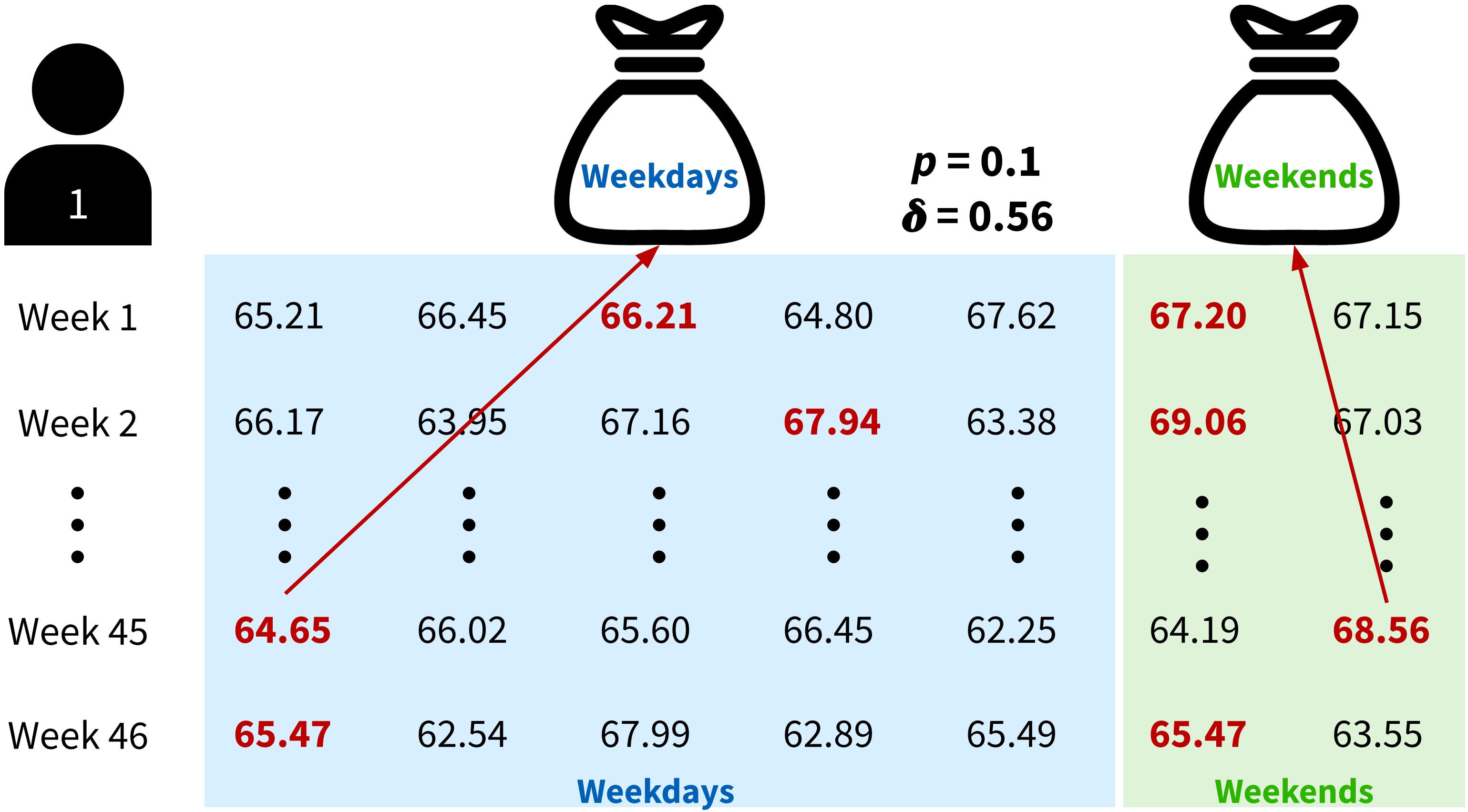

Within-Individual Sequential Sampling

Key characteristics:

- Weekend-weekday sampling done within-person entirely.
- Accounts for both **person-dependent** and **time-dependent** differences in HR.
- Pairs of weekend-weekday HR are randomly chosen from each individual's data, and the weekend and weekday HR values are from the same calendar week. **Importantly**, the weeks from which the data are sampled are in order of calendar week, where sampling begins from the individual's first calendar week and ends with their last calendar week.

Within-Individual Sequential Sampling

1

Weekdays

Weekends

Week 1	65.21	66.45	66.21	64.80	67.62	67.20	67.15
Week 2	66.17	63.95	67.16	67.94	63.38	69.06	67.03
Week 3	64.65	66.02	65.60	66.45	62.25	64.19	68.56
Week 4	64.65	66.02	65.60	66.45	62.25	64.19	68.56
⋮	⋮	⋮	⋮	⋮	⋮	⋮	⋮

Weekdays

Weekdays

Within-Individual
Sequential Sampling

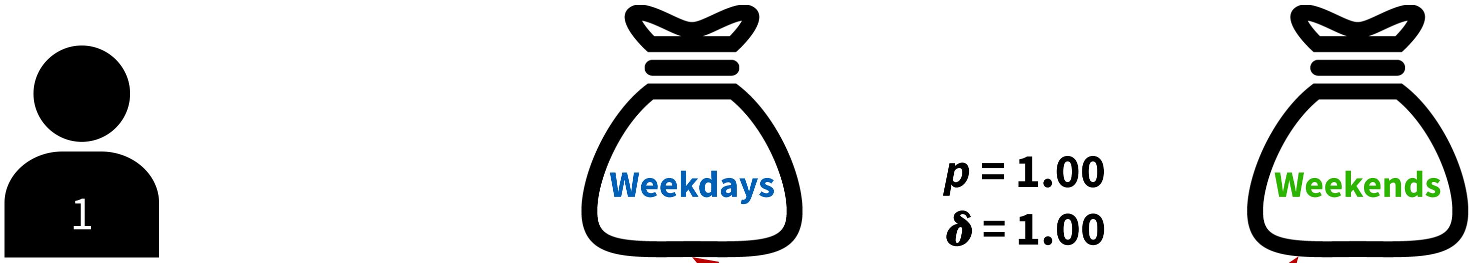

$p = 1.00$
$\delta = 1.00$

Week 1	65.21	66.45	66.21	**64.80**	67.62	**67.20**	67.15
Week 2	66.17	63.95	67.16	67.94	63.38	69.06	67.03
Week 3	64.65	66.02	65.60	66.45	62.25	64.19	68.56
Week 4	64.65	66.02	65.60	66.45	62.25	64.19	68.56
⋮	⋮	⋮	⋮	⋮	⋮	⋮	⋮

Weekdays

Weekdays

Within-Individual Sequential Sampling

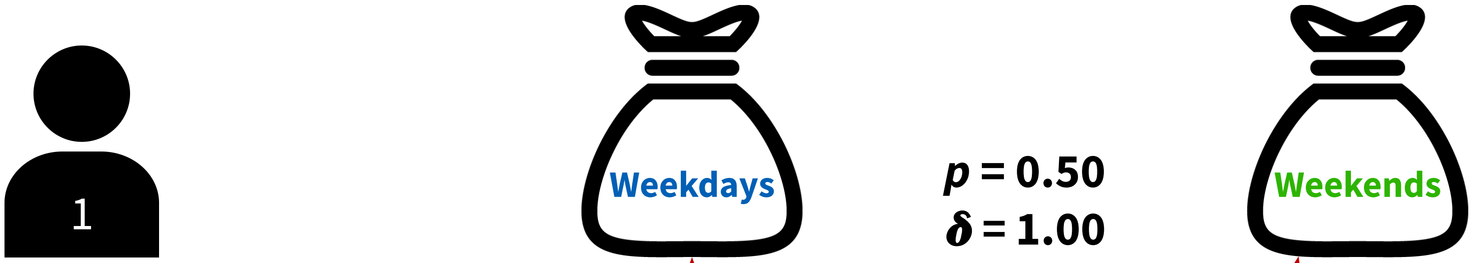

$p = 0.50$
$\delta = 1.00$

Week 1	65.21	66.45	66.21	**64.80**	67.62	**67.20**	67.15
Week 2	66.17	63.95	**67.16**	67.94	63.38	**69.06**	67.03
Week 3	64.65	66.02	65.60	66.45	62.25	64.19	68.56
Week 4	64.65	66.02	65.60	66.45	62.25	64.19	68.56
⋮	⋮	⋮	⋮	⋮	⋮	⋮	⋮
			Weekdays			Weekdays	

Within-Individual Sequential Sampling

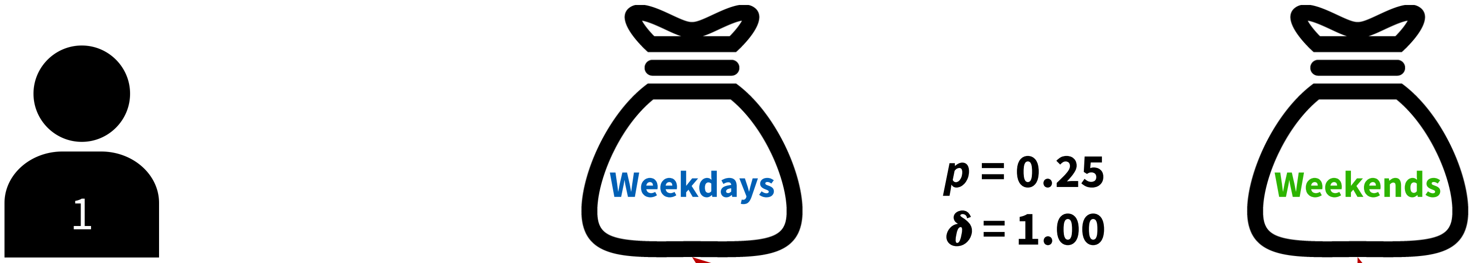

$p = 0.25$
$\delta = 1.00$

Week 1	65.21	66.45	66.21	**64.80**	67.62	**67.20**	67.15
Week 2	66.17	63.95	**67.16**	67.94	63.38	**69.06**	67.03
Week 3	64.65	66.02	65.60	66.45	**62.25**	64.19	**68.56**
Week 4	64.65	66.02	65.60	66.45	62.25	64.19	68.56
⋮	⋮	⋮	⋮	⋮	⋮	⋮	⋮

Weekdays

Weekdays

Within-Individual Sequential Sampling

1

Weekdays

$p = 0.38$
$\delta = 0.62$

Weekends

Week 1	65.21	66.45	66.21	**64.80**	67.62	**67.20**	67.15
Week 2	66.17	63.95	**67.16**	67.94	63.38	**69.06**	67.03
Week 3	64.65	66.02	65.60	66.45	**62.25**	64.19	**68.56**
Week 4	64.65	66.02	65.60	**66.45**	62.25	**64.19**	68.56
⋮	⋮	⋮	⋮	⋮	⋮	⋮	⋮

Weekdays

Weekdays
