# Supplementary material for: Testing the Impact of Intensive, Longitudinal Sampling on Assessments of Statistical Power and Effect Size Within a Heterogeneous Human Population: Natural Experiment Using Change in Heart Rate on Weekends as a Surrogate Intervention
Source: J Med Internet Res. 2025 May 21;27:e60284. doi: 10.2196/60284 (PMC12138295; doi:10.2196/60284)
Supplement: Multimedia Appendix 2 [file jmir_v27i1e60284_app2.docx]

| **Table 1.**  Mean and Standard Deviation for Each Sampling Method at *P*<.01. | | | |
| --- | --- | --- | --- |
| **Sampling Method** | **Mean (SD) Number of Samples Required for Significance** |  | **Mean (SD) Effect Size (*δ*) at Significance** |
| **Random** | 578.24 (519.44) |  | 0.14 (0.16) |
| **Temporal** | 594.85 (539.08) |  | 0.13 (0.14) |
| **Person-Matched** | 161.16 (131.60) |  | 0.12 (0.07) |
| **Temporal Person-Matched** | 142.02 (111.27) |  | 0.11 (0.08) |
| **Within-individual** | 14.27 (5.07) |  | 0.36 (0.44) |
| **Within-individual Temporal** | 14.32 (5.10) |  | 0.34 (0.40) |
| **Within-individual Sequential** | 15.12 (6.38) |  | 0.33 (0.42) |
