# Supplementary material for: Testing the Impact of Intensive, Longitudinal Sampling on Assessments of Statistical Power and Effect Size Within a Heterogeneous Human Population: Natural Experiment Using Change in Heart Rate on Weekends as a Surrogate Intervention
Source: J Med Internet Res. 2025 May 21;27:e60284. doi: 10.2196/60284 (PMC12138295; doi:10.2196/60284)
Supplement: Multimedia Appendix 3 [file jmir_v27i1e60284_app3.docx]

| **Table 1.** Median, IQR, mean, and standard deviation for each sampling method at *P*<.05. | | | | | |
| --- | --- | --- | --- | --- | --- |
|  | **Number of Samples Required for Significance** | |  | **Effect Size (*δ*) at Significance** | |
| **Sampling Method** | **Median (IQR)** | **Mean (SD)** |  | **Median (IQR)** | **Mean (SD)** |
| **Random** | 132.5  (26.00-390.00) | 273.74  (349.27) |  | 0.12  (0.07-0.27) | 0.17  (0.30) |
| **Temporal** | 133.0  (31.75-410.00) | 286.60  (383.71) |  | 0.12  (0.07-0.25) | 0.16  (0.24) |
| **Person-Matched** | 58.0  (20.00-118.25) | 85.27  (89.02) |  | 0.11  (0.07-0.18) | 0.14  (0.12) |
| **Temporal Person-Matched** | 50.0  (20.00-107.00) | 76.34  (76.04) |  | 0.10  (0.07-0.17) | 0.13  (0.12) |
| **Within-individual** | 9.0  (7.00-12.00) | 11.05  (3.82) |  | 0.45  (0.24-0.57) | 0.28  (0.39) |
| **Within-individual Temporal** | 9.0  (7.00-12.00) | 11.03  (3.87) |  | 0.4  (0.16-0.53) | 0.26  (0.37) |
| **Within-individual Sequential** | 9.0  (7.00-13.00) | 11.53  (4.82) |  | 0.41  (-0.03 to 0.55) | 0.25  (0.41) |
