# Supplementary material for: Testing the Impact of Intensive, Longitudinal Sampling on Assessments of Statistical Power and Effect Size Within a Heterogeneous Human Population: Natural Experiment Using Change in Heart Rate on Weekends as a Surrogate Intervention
Source: J Med Internet Res. 2025 May 21;27:e60284. doi: 10.2196/60284 (PMC12138295; doi:10.2196/60284)
Supplement: Multimedia Appendix 4 [file jmir_v27i1e60284_app4.docx]

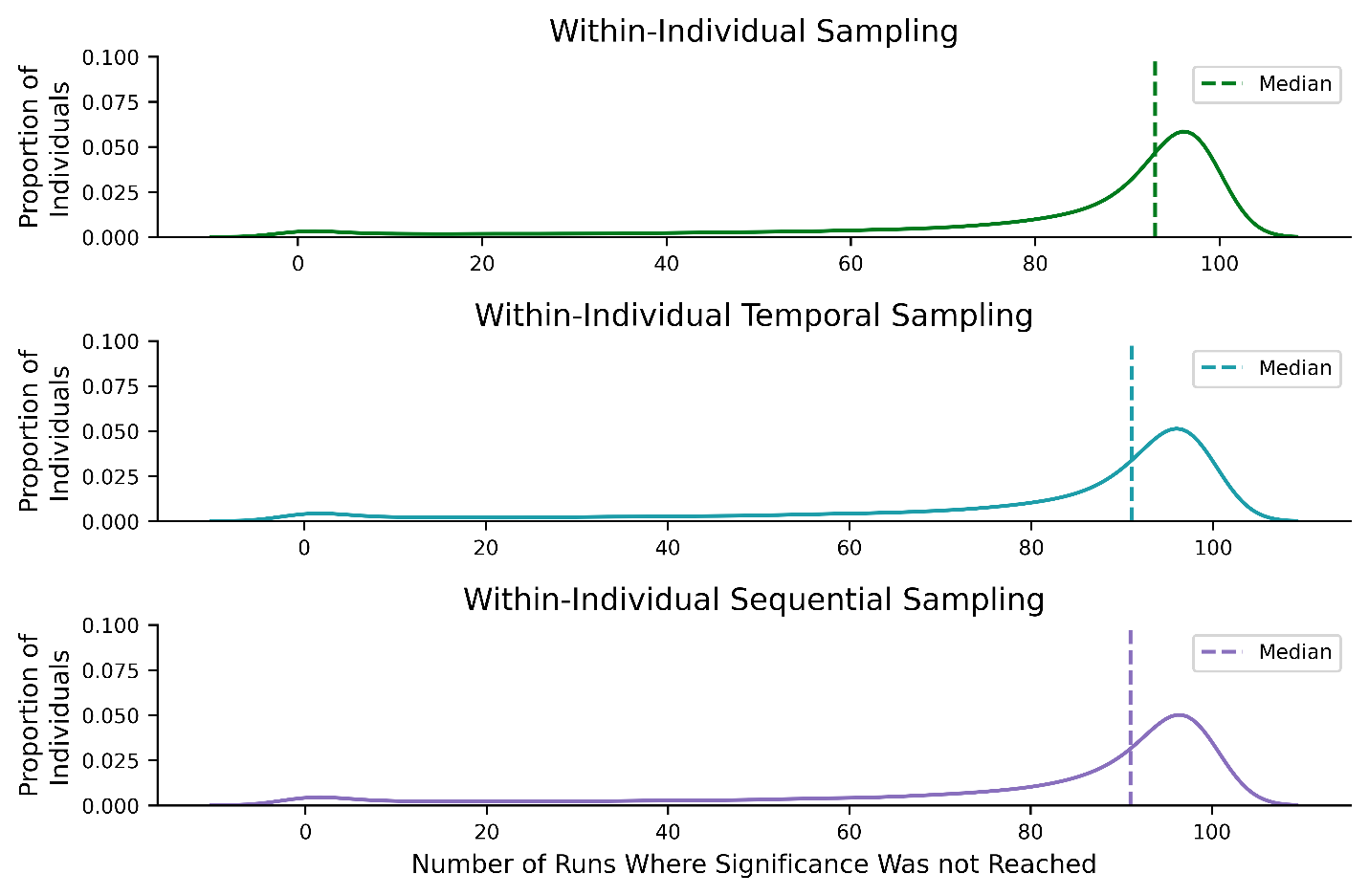


**C**

**B**

**A**

Figure 1. Distributions of number of runs where significance was not reached for (A) within-individual sampling, (B) within-individual temporal sampling, and (C) within-individual sequential sampling.
