# Supplementary material for: Testing the Impact of Intensive, Longitudinal Sampling on Assessments of Statistical Power and Effect Size Within a Heterogeneous Human Population: Natural Experiment Using Change in Heart Rate on Weekends as a Surrogate Intervention
Source: J Med Internet Res. 2025 May 21;27:e60284. doi: 10.2196/60284 (PMC12138295; doi:10.2196/60284)
Supplement: Multimedia Appendix 5 [file jmir_v27i1e60284_app5.docx]

| **Table 1.** Spearman Correlations Between Effect Size, Reaching Statistical Significance, and Data Availability. | | | |
| --- | --- | --- | --- |
|  | **Median effect size per individual**  **(*δ*)** | **Number of runs where significance was not reached per individual** | **Maximum weeks of data available per individual** |
| **Median effect size per individual**  **(*δ*)** | 1.000 | ***  -0.240 | ***  -0.462 |
| **Number of runs where significance was not reached per individual** | ***  -0.240 | 1.000 | ***  -0.234 |
| **Maximum weeks of data available per individual** | ***  -0.462 | ***  -0.234 | 1.000 |
| ****P*<.001. | | | |
